# Methodological framework for chromogenic mRNA detection using in situ hybridization chain reaction

**DOI:** 10.1007/s00418-026-02482-w

**Published:** 2026-05-06

**Authors:** Mitsuru Yashiro, Yousuke Tsuneoka, Yusuke Atsumi, Aki Makanae, Hiromasa Funato

**Affiliations:** 1 Department of Anatomy, Faculty of Medicine, Toho University, Tokyo, Japan; 2 Center for Research and Product Development, Nepa Gene Co., Ltd., Chiba, Japan; 3 International Institutes for Integrative Sleep Medicine (WPI-IIIS), University of Tsukuba, Ibaraki, Japan

**Keywords:** In situ HCR, Chromogenic staining, Regional marker, Single-molecule ISH, ISH–IHC combination

## Abstract

In situ hybridization chain reaction (HCR) is a highly sensitive method for visualizing single-molecule messenger RNA (mRNA) using self-assembling hairpin DNA pairs. However, conventional in situ HCR is primarily optimized for fluorescence detection, limiting its applicability in routine pathology and in tissues with strong autofluorescence. In this study, we established a chromogenic in situ HCR protocol using short hairpin DNA that achieves sensitivity comparable to fluorescent in situ HCR. To achieve efficient amplification and avoid steric hindrance, we adopted an indirect labeling strategy in which hairpin DNA conjugated with haptens (biotin, digoxigenin, or fluorescein) is detected using enzyme-conjugated streptavidin or antibodies. We validated this approach across various mouse tissues, including the brain, kidney, and liver, and on both slide-mounted and free-floating sections. The protocol enabled the clear visualization of low-abundance transcripts such as *Oxtr* and *Esr1*. Furthermore, we demonstrated the versatility of this approach by performing duplex chromogenic staining for two mRNA targets and by combining in situ HCR with immunohistochemistry to visualize mRNA and protein simultaneously. This chromogenic in situ HCR retains the high sensitivity of HCR and adds the practical advantages of bright-field detection, thereby expanding the utility of in situ HCR in histological research.

**Supplementary Information:**

The online version contains supplementary material available at 10.1007/s00418-026-02482-w.

## Introduction

In situ hybridization (ISH) is a major technique for visualizing mRNA localization in tissues. In ISH, the specific nucleic acid probes with a sequence complementary to the target mRNA or other nucleic acid are hybridized in the tissue, and the probe can be visualized using various methods. As probe hybridization to the target mRNA occurs in a sequence-specific manner, ISH can be applied to most species, provided that the target sequences are known. Conventional ISH uses long complementary RNA/DNA probes labeled with small molecules such as biotin, digoxigenin (DIG), and fluorescein, and these molecules are visualized through immunohistochemical methods. Although conventional ISH is still the most widely used method, many highly sensitive ISH methods have been developed over the past quarter century (Veselinyová et al. 2021; Le et al. 2022; Gerber et al. 2023; Hong 2025).

In ISH, various issues are known to affect accurate detection of mRNA in tissue samples. Among them, sensitivity is a major concern, and signal amplification techniques are necessary to visualize mRNA in many cases. Labeling with alkaline phosphatase (AP) and signal amplification through AP substrate reactions is one of the most widely used methods, called chromogenic ISH (CISH). Methods using peroxidase (POD) and its substrates are also employed for CISH, but their amplification efficiency is generally lower than that of AP. POD is also employed in amplification techniques such as catalyzed reporter deposition, also known as tyramide signal amplification (TSA), where many activated tyramide compounds can be attached near the enzyme (Schmidt et al. 1997; Wanner et al. 1997; Yang et al. 1999). To date, methods that amplify the nucleic acid itself, rather than the detection dyes via enzyme reaction, have emerged as distinct approaches to signal enhancement for ISH. These approaches include hybridization chain reaction (HCR) (Choi et al. 2010), rolling circle amplification (RCA) (Larsson et al. 2010), and branched DNA techniques (Player et al. 2001). While their amplification strategies differ, they all utilize mRNA and probe DNA as a scaffold for DNA amplification, resulting in the hybridization of numerous dye-labeled readout DNA probes.

HCR is a self-catalytic double-stranded DNA elongation process, in which the hybridization of single hairpin DNA to the initiator DNA opens the hairpin structures, and exposed single-stranded DNA hybridizes with other hairpin DNA. Hybridization triggers the subsequent hybridization of hairpin DNA, resulting in the elongation of double stranded DNA (Dirks and Pierce 2004). In a HCR, the hairpin DNA can be directly labeled with dyes, thereby functioning as the readout signal itself. The RCA system utilizes a circular probe as a template for amplification by DNA polymerase, resulting in the synthesis of a long single-stranded DNA that is the target of dye-labeled readout probe (Larsson et al. 2004). In the branched DNA system, single-stranded DNA is repeatedly hybridized through branching structures, resulting in a large number of dye-labeled readout probe DNA binding to the branched DNA structures that extend from the target RNA. This technique has been adopted in RNAscope and ViewRNA (Wang et al. 2012). While all of these are highly sensitive and enable the visualization of single-copy mRNA, the ISH using HCR amplification (in situ HCR) offers several advantages, including a simple, low-step workflow and its high cost-efficiency.

In situ HCR consists of two major steps: hybridization of probes with HCR initiating sequences and signal amplification by hairpin DNA. One of the advantages of in situ HCR is that it requires minimal hands-on time owing to its simple procedure (Choi et al. 2014). Moreover, HCR only proceeds when paired probes hybridize precisely at their target sites, which minimizes nonspecific reactions (Choi et al. 2018). We have previously developed an in situ HCR method using short hairpin DNA, which improved probe permeability and simplified the overall protocol (Tsuneoka and Funato 2020). Despite its advantages, in situ HCR is primarily optimized for fluorescence detection of messenger RNA (mRNA), and there have been limited published reports demonstrating its chromogenic visualization (Matsuzaki et al. 2025).

Fluorescent staining enables the simultaneous observation of multiple molecules, whereas chromogenic visualization is limited in the number of distinguishable dyes. Nevertheless, chromogenic visualization remains a widely used technique owing to the following advantages. First, chromogenic dyes are generally resistant to photobleaching and can be observed over extended periods when sealed with an organic mounting medium. Second, the equipment required for observing chromogenic dyes is simpler and more compact than that used for fluorescence imaging, and the imaging process itself is faster, making it well-suited for routine applications such as pathological examinations. Additionally, chromogenic visualization avoids the issue of autofluorescence of biological samples, which sometimes interferes with signal clarity in fluorescence-based methods (Poulsen et al. 2013).

**Fig. 1:**
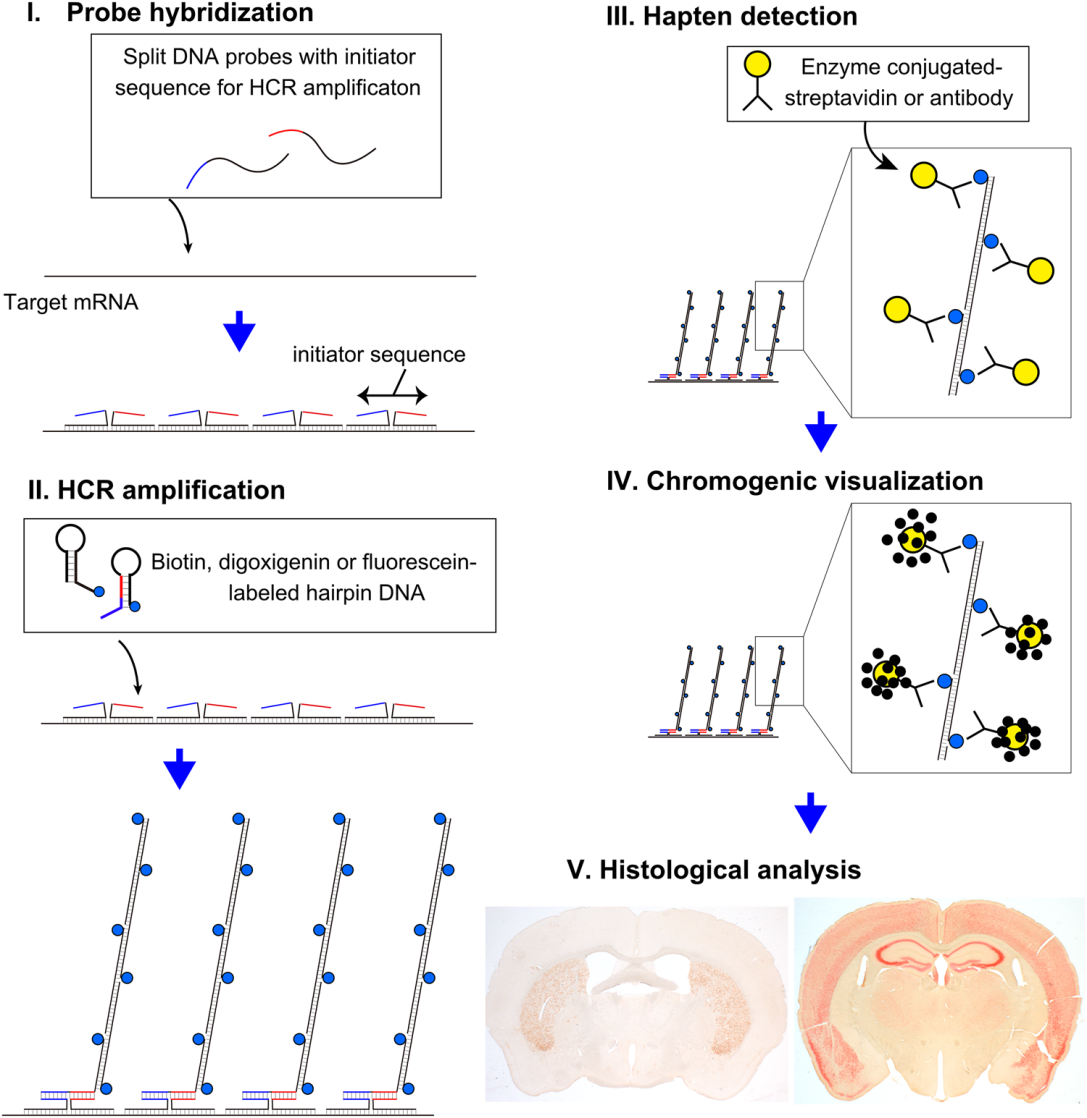
Schematic of chromogenic HCR staining using hapten-labeled hairpin DNA. Probe hybridization is achieved through a split-probe system, in which the HCR initiator sequence is formed only when specific hybridization to the target mRNA occurs. For HCR amplification, hairpin DNA labeled with either biotin, digoxigenin (DIG), or fluorescein is used. Through the HCR reaction, numerous haptens accumulate on the target mRNA. Subsequently, the haptens are detected by enzyme-conjugated specific antibodies or streptavidin, and chromogenic visualization is performed via the associated enzymatic reaction

In this study, we developed an in situ HCR-based chromogenic staining protocol with sensitivity comparable to that of fluorescent in situ HCR. Achieving highly efficient amplification of HCR requires optimized labeling features of short hairpin DNA (Tsuneoka and Funato 2020). To avoid steric hindrance that interferes with the HCR, the dyes conjugated to the hairpin DNA should possess a compact chemical structure, and it is preferable to spatially separate the dyes from the DNA. In addition, since heating denaturation of hairpin DNA is required for stable HCR amplification and the chromogenic enzymes would be inactivated by heat, the direct conjugation of chromogenic enzymes to the hairpin DNA is not a practical approach. Therefore, we adopted indirect labeling of the chromogenic enzyme, as shown in Fig. 1. Hairpin DNA was labeled with small molecules such as biotin, DIG, or fluorescein. Detection of these molecules was performed by immunohistochemistry using streptavidin or antibodies conjugated to enzymes such as POD or AP, followed by chromogenic development with appropriate substrates. These approaches enabled chromogenic visualization in diverse tissues. Furthermore, we showed the multiplex staining of the chromogenic ISH for two target mRNAs, as well as the combination of chromogenic ISH combined with immunohistochemistry.

## Materials and methods

### Animals

Breeding pairs of C57BL/6 J mice were purchased from SLC and CLEA Japan. Mice were raised in our breeding colony under controlled conditions (12-h light/dark cycle, lights on at 8:00 a.m., 23 ± 2 °C, 55 ± 5% humidity, and ad libitum access to water and food) and used at 12–20 weeks of age. Virgin female mice and primiparous mothers were used to compare expression level of *Oxtr* mRNA. Mother mice were sampled on postpartum day 7 after their first delivery. For experiments examining *Esr1* expression in the brain, virgin females were also used. All other experiments were performed using 12–20-week-old virgin male mice. All animal experiments were conducted in accordance with the guidelines for Animal Experiments of Toho University and were approved by the Institutional Animal Care and Use Committee of Toho University (approval protocol ID no. 24-557) prior to initiating this study.

Mice were deeply anesthetized with isoflurane and then transcardially perfused with 4% paraformaldehyde (PFA) in phosphate-buffered saline (PBS) for 5 min at a rate of 10 ml/min. The brains, stomach, liver, kidney, and testes were dissected, and postfixed in 4% PFA at 4 °C overnight, followed by cryoprotection in 30% sucrose in PBS for 2 days, embedded in Surgipath (FSC22, Leica Biosystems, Nussloch, Germany), and stored at −80 °C. The frozen tissues were cryosectioned at a thickness of 40 µm for the brains and 10 µm for other tissues. The brain sections were stored in the antifreeze solution (30% glycerol, 30% ethylene glycol, 0.02 M phosphate buffer), and other sections were mounted on adhesive-coated glass slides (Platinum Pro, Matsunami, Osaka, Japan) and stored at −20 °C until use.

### Preparation of probes and hairpin DNA labeling

The probes for in situ HCR were designed to minimize off-target complementarity using a homology search by NCBI BLASTn (https://blast.ncbi.nlm.nih.gov/Blast.cgi), and they were designed to have split-initiator sequences with an mRNA binding site (Supplementary Table S1). The number of target sites per single mRNA varied from 5 to 21 on the basis of their abundance in the tissue. The number of hairpin DNA pairs designed for each target mRNA is summarized in Supplementary Table 2. The DNA probes were synthesized as standard desalted oligos (Integrated DNA Technologies, Coralville, IA, USA) and used without additional purification procedure. The hairpin DNA was synthesized with a C12 amino-linker at the 5′ end (Integrated DNA Technologies or Tsukuba Oligo Services, Ibaraki, Japan), and the amine bases were conjugated with the succinimidyl ester of tag molecules (DIG, biotin, fluorescein, SaraFluor488, ATTO550, or Cy5). The hairpin DNA was purified by denaturing polyacrylamide gel electrophoresis using 20% polyacrylamide gels. The detailed procedures for DNA purification are described in our previous study (Tsuneoka and Funato 2020).

### Probe hybridization and HCR amplification

Probe hybridization and HCR amplification procedures were similar to those previously described (Tsuneoka and Funato 2020; Tsuneoka et al. 2022). The free-floating sections or mounting sections were washed with PBS containing 0.2% Triton (PBST) and immersed in 3% H_2_O_2_ methanol for 10 min, followed by PBST washing for 5 min twice. After washing, the sections were prehybridized for 5 min at 37 °C in a hybridization buffer containing 10% dextran sulfate, 0.5× SSC, 0.1% Tween 20, 50 µg/ml heparin, and 1× Denhardt’s solution. The sections were treated with another hybridization solution containing a mixture of 10 nM probes and incubated overnight at 37 °C. The control experiment was performed without probes. In addition, for *Penk*, we examined samples pretreated with RNase A solution (20 µg/ml in PBST, Sigma-Aldrich, St. Louis, MO, USA; R6513) at 37 °C for 30 min before hybridization. In the in situ HCR, signal generation requires simultaneous hybridization of two split initiator probes to the 5′ and 3′ halves of a 50-nucleotide target sequence. This design suppresses nonspecific hybridization of hairpin DNA because the signal is only produced when both probes hybridize correctly (Choi et al. 2018, also see Fig. 1). To further validate this requirement for dual-probe hybridization, we performed experiments using “unpaired probes” in which the 5′ and 3′ halves were mismatched but shared the same initiator sequence. Unpaired probe combinations included *Penk*–*Esr1*, *Oxtr*–*Drd1*, and *Drd2*–*Lrp*. After hybridization, the sections were washed three times for 10 min in 0.5× SSC containing 0.1% Tween 20 at 37 °C. For fluorescent staining, the sections were bleached by a light-emitting diode ( LED) illuminator TiYO (Nepagene Co., Ltd., Chiba, Japan) for 60 min in PBST to quench autofluorescence after washing.

For HCR amplification, one or two of the following hairpin DNA pairs were used: S23, S41, S45, S72, or A161 (Table 1). For the combination of *Penk* probes and the A161 hairpin DNA, we also performed no-hairpin DNA control experiments. These hairpin DNA solutions were separately heated to 95 °C for 1 min and then gradually cooled to 65 °C for 15 min and to 25 °C for 40 min before use. The sections were incubated in an amplification buffer (10% dextran sulfate in 8× SSC, 0.2% Triton X-100, and 100 mM MgCl_2_) for 5 min and then immersed in another amplification buffer containing 60 nM hairpin DNA pairs for 2 h at 25 °C. The samples were washed with PBST three times for 5 min. For fluorescent staining, the amplification buffer was supplemented with Hoechst 33,342 (1 µg/ml, Dojindo, Kumamoto, Japan; H342), and the slides were coverslipped with VECTASHIELD Vibrance (Vector Laboratory, Burlingame, CA, USA; H-1700).

**Table 1:** Summary of reaction conditions for each target mRNA

Target mRNA	Hairpin ID	Label	Antibody or streptavidin	Concentration	Incubation time	Chromogenic substrates
Single-plex conditions						
*Drd2*	S23	DIG	AP-conjugated anti-DIG antibody	1/1000	Overnight	ImmPACT Vector Red
*Esr1*	A161	DIG	AP-conjugated anti-DIG antibody	1/1000	Overnight	ImmPACT Vector Red
*Oxtr*	S41	Biotin	POD-conjugated streptavidin	1/1000	0.5 h	ImmPACT DAB
*Penk*	A161	Biotin	POD-conjugated streptavidin	1/1000	0.5 h	ImmPACT DAB
		DIG	POD-conjugated anti-DIG antibody	1/1000	Overnight	ImmPACT DAB
		Fluorescein	POD-conjugated anti-fluorescein antibody	1/1000	Overnight	ImmPACT DAB
		Biotin	AP-conjugated streptavidin	1/1000	0.5 h	ImmPACT Vector Red
		DIG	AP-conjugated anti-DIG antibody	1/1000	Overnight	ImmPACT Vector Red
		Fluorescein	AP-conjugated anti-fluorescein antibody	1/1000	Overnight	ImmPACT Vector Red
*Periostin*	S72	Biotin	POD-conjugated streptavidin	1/250	1 h	ImmPACT DAB
Duplex conditions						
*Albumin*	S45	Biotin	POD-conjugated streptavidin	1/250	1 h	ImmPACT DAB
*Cyp2e1*	S72	Fluorescein	AP-conjugated anti-fluorescein antibody	1/500	Overnight	BCIP/NBT
*Drd1*	S41	Biotin	POD-conjugated streptavidin	1/1000	0.5 h	ImmPACT DAB
*Esr1*	A161	Biotin	POD-conjugated streptavidin	1/1000	1 h	ImmPACT DAB
*Lrp2*	S23	Fluorescein	AP-conjugated anti-fluorescein antibody	1/500	Overnight	BCIP/NBT
						ImmPACT Vector Red
*Penk*	S23	DIG	AP-conjugated anti-DIG antibody	1/1000	Overnight	ImmPACT Vector Red
*Umod*	A161	DIG	POD-conjugated anti-DIG antibody	1/500	Overnight	ImmPACT DAB
*Vglut1*	S10	Fluorescein	AP-conjugated anti-fluorescein antibody	1/500	Overnight	ImmPACT Vector Red

### Chromogenic visualization

**Fig. 2:**
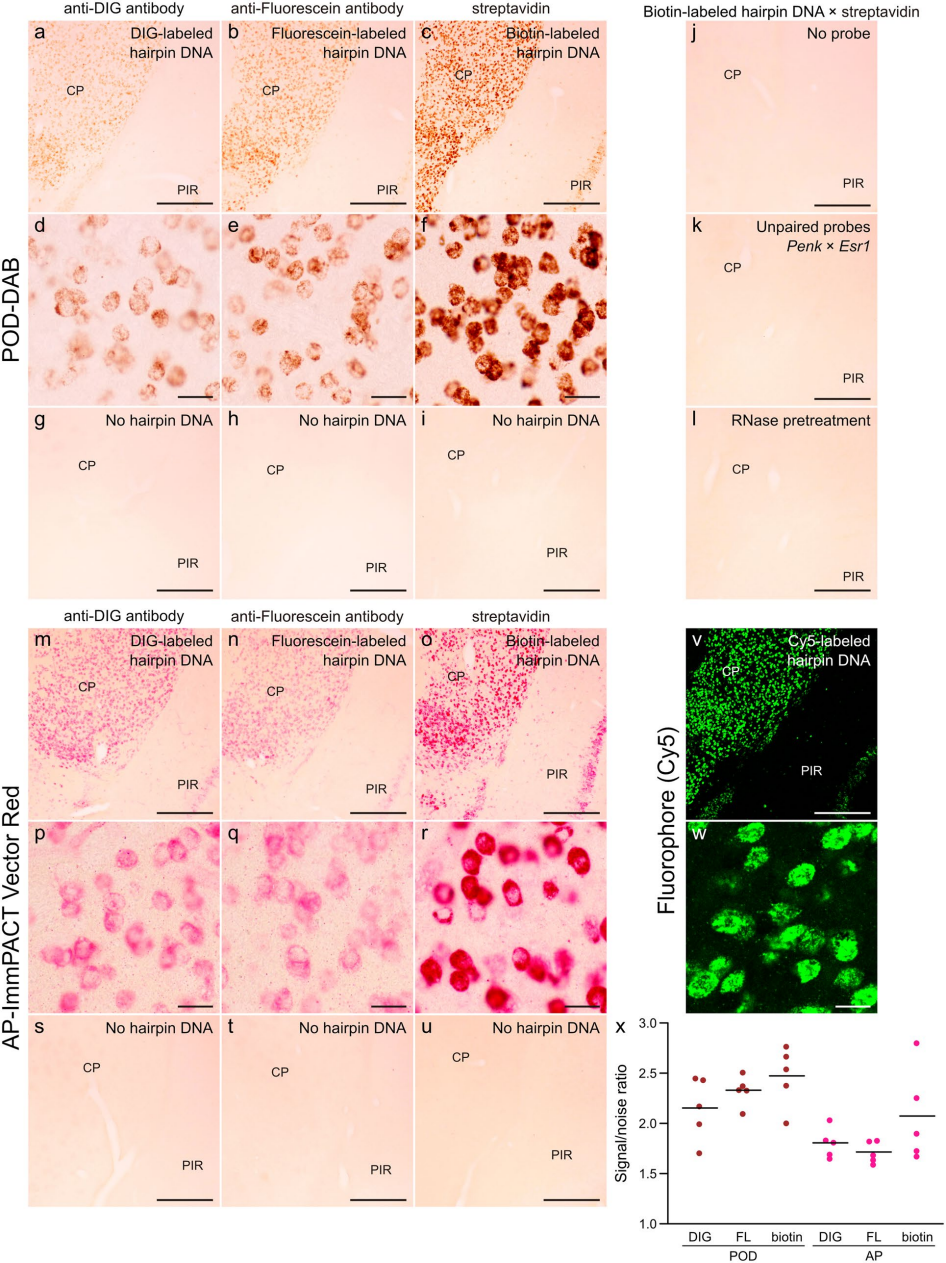
Comparison of hapten and enzyme combinations for chromogenic HCR staining in floating sections of the mouse brain. In situ HCR for *Penk* mRNA in the mouse brain using hapten-labeled hairpin DNA. **a**–**u** Chromogenic in situ HCR staining. The enzyme–chromogen combinations were POD–DAB (**a**–**l**) and AP–ImmPACT Vector Red (**m**–**u**). **v**, **w** Fluorescent in situ HCR staining. **a**, **d**, **m**, **p** Hairpin DNA labeled with DIG, and detected by anti-DIG antibody. **b**, **e**, **n**, **q** Hairpin DNA labeled with fluorescein, and detected by anti-fluorescein antibody. **c**, **f**, **o**, **r** Hairpin DNA labeled with biotin and detected by streptavidin. **g**–**i**, **s**–**u** No hairpin DNA controls. **j** No-probe control. **k** Unpaired probe control of *Penk* and *Esr1*. **l** RNase pretreatment control. **x** Comparison of signal/noise ratio (*n* = 5 for all). Bars represent means. Tissue sections shown in **a**–**f**, **m**–**r**, and **v**–**w** were prepared from the same mouse. Scale bars: 400 μm (**a**–**c**, **g**–**o**, **s**–**v**) and 25 μm (**d**–**f**, **p**–**r**, **w**)

After HCR amplification, the sections were subjected to an immunohistochemical procedure. In the sections labeled by biotinylated hairpin DNA, tissue sections were immersed in the POD- or AP-conjugated streptavidin (Jackson ImmunoResearch, West Grove, PA, USA; 016-030-084 or 016-050-084) in PBST for 30 min at 37 °C. In the sections labeled by DIG- or fluorescein-conjugated hairpin DNA, the sections were blocked with 0.8% Block Ace (Dainihon-Seiyaku, Osaka, Japan) in PBST for 30 min. Then, one AP-conjugated sheep anti-DIG antibody (Fab fragment, Roche, Basel, Switzerland; 11093274910), AP-conjugated sheep anti-fluorescein antibody (Fab fragment, Roche, Basel, Switzerland; 11426338910), POD-conjugated mouse anti-DIG antibody (Jackson ImmunoResearch, West Grove, PA, USA; 200-032-156), or POD-conjugated mouse anti-fluorescein antibody (Jackson ImmunoResearch, West Grove, PA, USA; 200-032-037) in 0.4% BlockAce/PBST was applied to the sections and incubated at 4 °C overnight. The antibody concentrations for each condition are summarized in Table 1, and the list of antibodies is presented in Table 2. For all anti-hapten antibodies and streptavidins, the absence of signal was confirmed in control conditions in which the in situ HCR probes or hairpin DNA was omitted (Fig. 2).

**Table 2:** Antibody information

Antibody	Catalog no.	RRID	Supplier
Antibodies for haptens			
Anti-DIG mouse antibody, POD conjugates	200-032-156	AB_2339011	Jackson ImmunoResearch, West Grove, PA, USA
Anti-DIG sheep antibody, AP conjugates	11093274910	AB_514497	Roche, Basel, Switzerland
Anti-fluorescein mouse antibody, POD conjugates	200-032-037	AB_2314402	Jackson ImmunoResearch, West Grove, PA, USA
Anti-fluorescein sheep antibody, AP conjugates	11426338910	AB_2734723	Roche, Basel, Switzerland
Primary antibody			
Anti-ERα rabbit antibody	06-935	AB_310305	Millipore, Billerica, MA, USA
Secondary antibodies			
Anti-rabbit horse antibody, POD conjugates	MP-7401	AB_2336529	Vector Laboratories, Burlingame, CA, USA
Anti-rabbit donkey antibody, AlexaFluor647 conjugates	ab150063	AB_2687541	Abcam, Cambridge, MA, USA
Streptavidin			
Streptavidin POD conjugates	016-030-084	AB_2337238	Jackson ImmunoResearch, West Grove, PA, USA
Streptavidin AP conjugates	016-050-084	AB_2337239	Jackson ImmunoResearch, West Grove, PA, USA

The sections were washed with PBST three times for 5 min for the POD reaction or with Tris-buffered saline with 0.1% Tween 20 three times for 5 min for the AP reaction. Then, the sections were visualized by the substrate solution as indicated in Table 1, according to the manufacturer’s instructions. For double staining, a cocktail of two antibodies conjugated to distinct enzymes was employed instead of a single antibody solution. Then, the POD substrate was developed first, followed by the AP substrate. In the color development of the AP substrate, endogenous AP was inhibited by adding 2 mM levamisole in the substrate solution. Part of the sections were counterstained by hematoxylin (Muto Pure Chemicals, Tokyo, Japan; Type M 30141). The sections were dehydrated with ethanol, cleared by xylene, and then coverslipped by Marinol (Muto Pure Chemicals, Tokyo, Japan; 20093).

### Double staining of in situ HCR and immunohistochemistry

For double staining of in situ HCR and immunohistochemistry, the procedures for probe hybridization and HCR amplification were the same as single staining. The probes for *Esr1* mRNA had split initiator sequence of A161 hairpin, and A161 hairpin pairs were conjugated with biotin for chromogenic detection, or with SaraFluor488 for fluorescent detection. After HCR amplification, the biotin-labeled sections were immersed in the POD-conjugated streptavidin. Then, the sections were visualized by DAB. The DAB-stained sections were immersed in 0.3% H_2_O_2_ methanol for 10 min to quench POD of streptavidin. After washing, the sections were blocked with 0.8% BlockAce in PBST for 30 min. Then, rabbit anti-ERα antibody (1: 20,000, Millipore, Billerica, MA, USA; C1355) in 0.4% BlockAce/PBST was applied to the sections and incubated at 4 °C overnight. The specificity of the anti-ERα antibody was validated by knockdown experiments, which confirmed the loss of immunoreactivity upon target depletion (Sano et al. 2013, 2016). The sections were washed with PBST three times for 5 min and incubated in POD polymer-conjugated horse anti-rabbit antibody (Vector laboratory, Burlingame, CA, USA; MP-7401) or AlexaFluor647-conjugated donkey anti-rabbit antibody (Abcam, Cambridge, MA, USA; ab150063) for 60 min at room temperature.

For chromogenic visualization, purple POD substrate (ImmPACT VIP substrate kit, Vector laboratories, Burlingame, CA, USA; SK-4605) was used according to the manufacturer’s instructions. After washing, coverslipping was performed in the same manner as for the single staining.

### Microscopy

Wide-field view of histological sections was obtained by a Nikon AZ100 universal zoom microscope (Nikon Instruments, Tokyo, Japan) equipped with a CMOS camera (Imaging Source, Charlotte NC, USA; DFK33UX178, 3072 × 2048 pixels). Optical photomicrographs were obtained using an Olympus BX50 microscope equipped with a digital camera (Canon, Tokyo, Japan; EOS Kiss X7, 3456 × 2304 pixels). Fluorescence photomicrographs with 16-bit color and 0.314 μm/pixel resolution were obtained using a Nikon Eclipse Ni microscope equipped with the A1R confocal detection system using 20×/0.75 NA objective lenses (Nikon Instruments, Tokyo, Japan). Tiled images were captured and automatically stitched by NIS-Elements C software version 5.21 (Nikon Instruments, Tokyo, Japan). All fluorescence images were processed solely by adjusting contrast, and identical adjustments were applied to all images presented within the same figure.

Image quantification was performed using ImageJ (version 1.54r; NIH, USA). To compare signal-to-noise ratios across different haptens and labeling enzymes, we quantified signals in *Penk* mRNA-stained sections. Microscope images were converted to grayscale, and gray values were measured in three 132 × 132 µm regions per section. Regions in the cerebral cortex that exhibit minimal *Penk* expression were used to define noise, whereas regions with strong, uniform *Penk* expression in the caudate putamen (CP) were used to define signal. The background gray value was defined as the gray value measured in a region without tissue. The signal-to-noise ratio was calculated as (mean signal value − background value)/(mean noise value − background value). For comparison with fluorescence staining, we quantified puncta number per cell for *Oxtr*, *Drd2*, *Periostin*, and *Esr1*, which exhibit relatively low expression levels and allow individual puncta to be resolved. When cell boundaries were unclear or cells were closely apposed, the midpoint between nuclei was regarded as the cell boundary. Data were obtained from two mice for *Esr1* and from three or more mice for each of the other targets. Puncta counts per cell were compared between chromogenic and fluorescence staining using Welch’s* t*-test, which does not assume equal variances. For *Oxtr*, the number of positive cells was also examined. Images were acquired from an 854 × 366 µm region in the central part of the medial preoptic area (MPOA) for each mouse, and cells with two or more puncta per cell were regarded as positive. Welch’s *t-*test was used to compare *Oxtr*-positive cell counts and puncta counts between virgin and mother mice.

## Results and discussion

### Single-plex chromogenic HCR staining

As illustrated in the experimental schematic in Fig. 1, probe pairs hybridize to the target mRNA sequence, thereby forming HCR initiators on the mRNA. HCR was subsequently performed using hairpin DNA conjugated with haptens, such as biotin, resulting in the localized accumulation of these haptens around the mRNA. Following the HCR reaction, POD- or AP-conjugated streptavidin was applied to the biotin-labeled sections, and POD- or AP-conjugated anti-hapten antibody was applied to the DIG or fluorescein-labeled sections. Finally, color development by POD and AP substrate enabled the visualization of mRNA in the tissue.

First, we investigated how different combinations of haptens and enzymes affect staining behavior on floating sections of mouse brains. As shown in Fig. 2, *Penk* mRNA was successfully detected in the mouse brain tissue sections using chromogenic in situ HCR, regardless of whether the hairpin DNA was labeled with DIG, fluorescein, or biotin. These signals were not observed when only probes and antibodies were reacted without hairpin DNA, confirming the specificity of the antibodies (Fig. 2g–i and s–u). All labeling strategies yielded a similar and specific distribution of *Penk* mRNA in the CP and piriform cortex (PIR), consistent with the expression pattern observed in the fluorescent in situ HCR staining (Fig. 2v,w) and previous reports (Turchan et al. 1997; Lobo et al. 2006; Labouesse et al. 2018). Such *Penk* expression distributions were not observed in either the no-probe control, the unpaired-probe control (targeting *Penk* in the first half and *Esr1* in the second half probes), or RNase-pretreated sections (Fig. 2j–l), suggesting that the observed chromogenic staining reflects specific *Penk* expression. However, the signal intensity varied among the haptens. DIG and fluorescein hairpin DNA produced comparable signals, while the biotin–streptavidin combination produced the strongest signals when detected with either POD (Fig. 2a–f) or AP (Fig. 2m–r). In addition, streptavidin-based detection provided a slightly higher signal-to-noise ratio than antibody-based detection, regardless of the labeling enzyme (Fig. 2x). Such differences in staining intensity among haptens are likely attributable to affinity of tags and variations in immunohistochemical detection methods. Although the avidin–biotin complex (ABC) method is another approach for detecting biotin conjugate, the signal tends to be much less intense when using the ABC method than the streptavidin–biotin method in chromogenic HCR, consistent with a previous report (McQuaid and Allan 1992). POD-polymer-conjugated streptavidin was sometimes used in immunohistochemical detection to enhance signal intensity, but it shows little difference in signal intensity compared with streptavidin conjugated with POD monomer (data not shown). These observations suggest that detection sensitivity is influenced not only by the differences in affinity for the haptens but also by factors such as tissue permeability and molecular size of the antibodies or streptavidin used for readout. Given its high sensitivity, the streptavidin–biotin staining method is often the preferred choice in chromogenic HCR staining. Nonetheless, in cases where endogenous biotin leads to unavoidable background, alternative tags such as DIG or fluorescein may offer more reliable detection.

**Fig. 3:**
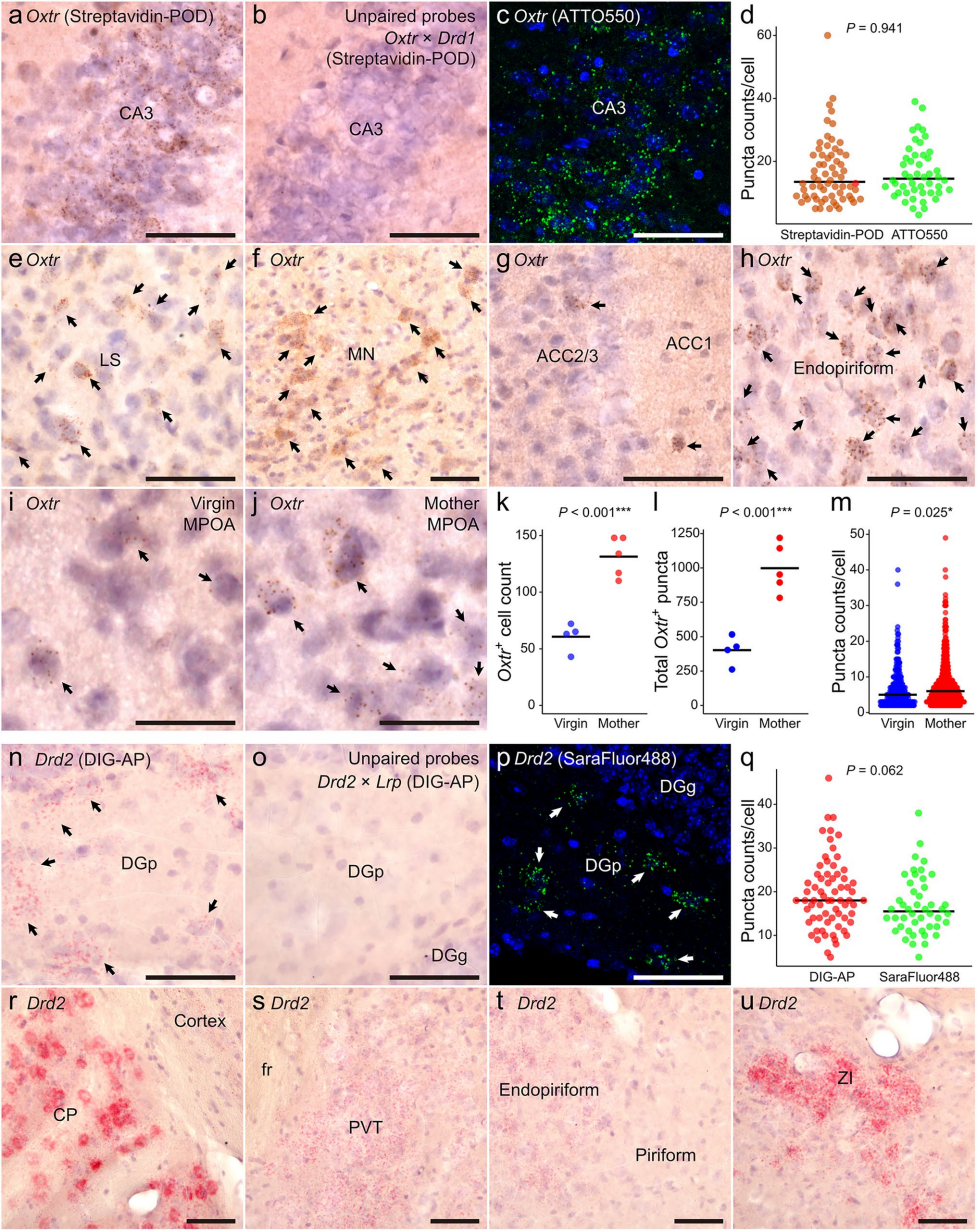
Representative images of chromogenic HCR staining for *Oxtr* and *Drd2* mRNA in the mouse brain. **a**, **c** *Oxtr* mRNA in the CA3 region of the hippocampus. **a** HCR was performed using biotin-conjugated hairpin DNA, bound by POD-conjugated streptavidin, and visualized by DAB substrate. **b** Unpaired probe control of *Oxtr* and *Drd1*. **c** HCR was performed using ATTO550-conjugated hairpin DNA. **d** Comparison of puncta counts/cell between chromogenic and fluorescent HCR staining of *Oxtr*. **e**–**h** *Oxtr* Expression in the lateral septum (LS) (**e**), magnocellular nucleus (MN) (**f**), anterior cingulate cortex (ACC), layer 1 and 2/3 (**g**), and endopiriform nucleus (**h**). **i**–**m** *Oxtr* Expression difference between virgin female (*n* = 4) and mother (*n* = 5) in the medial preoptic area (MPOA). **i** Representative expression in virgin MPOA. **j** Representative expression in mother MPOA. **k** Difference in *Oxtr*-positive cell number. **l** Difference in total *Oxtr*-positive puncta. **m** Difference in *Oxtr* puncta for individual cells (virgin: 243 cells; mother: 658 cells). **n**, **p** *Drd2* mRNA in the dentate gyrus (DG). **n** HCR was performed using DIG-conjugated hairpin DNA, bound by AP-conjugated anti-DIG antibody, and visualized by VectorRed substrate. **o** Unpaired probe control of *Drd2* and *Lrp*. **p** HCR was performed using Cy5-conjugated hairpin DNA. Arrowheads indicate the *Drd2* mRNA-positive cells. **q** Comparison of puncta counts/cell between chromogenic and fluorescent HCR staining of *Drd2*. **r**–**u** *Drd2* expression in caudate putamen (CP) (**r**), paraventricular thalamic nucleus (PVT) (**s**), endopiriform nucleus (**t**), and zona incerta (ZI) (**u**). Bars represent median values (**d**, **m**, **q**) and means (**k**, **l**). Scale bars: 25 μm (**i**–**j**), 50 μm (others). *DGp* dentate gyrus, polymorphic layer, *DGg* dentate gyrus, granule cell layer, *fr* fasciculus retroflexus

Next, we attempted to visualize genes with low expression levels using chromogenic HCR staining in the mouse brain (Fig. 3). *Oxtr* mRNA was stained using biotinylated hairpin DNA combined with POD-conjugated streptavidin. Numerous granular brown signals were observed in the hippocampal CA3 region (Fig. 3a), consistent with the distribution observed in fluorescent HCR staining of *Oxtr* mRNA (Fig. 3c). However, no signal was observed with the unpaired probe targeting *Oxtr* and *Drd1*. The distribution of *Oxtr* localized in the CA3 region is further supported by the observation from OXTR-VENUS mice (Lin et al. 2017). There was no significant difference in puncta counts per cell between chromogenic and fluorescence staining in the CA3 region (Fig. 3d). In addition, we examined *Oxtr* expression in the lateral septum (LS), medial nucleus of the hypothalamus (MN), anterior cingulate cortex (ACC), and endopiriform nucleus, where *Oxtr* expression has been previously reported (Yoshimura et al. 1996; Ostrowski 1998; Tsuneoka et al. 2013; Menon et al. 2018; Sharma et al. 2019; Tsuneoka and Funato 2020; Hidema et al. 2023; Li et al. 2024), and observed specific expression patterns in these regions (Fig. 3e–h). We also examined sections from the mouse medial preoptic area (MPOA), where *Oxtr* expression is known to increase postpartum (Meddle et al. 2007). In the MPOA, mothers showed higher numbers of *Oxtr*-positive cells, higher total *Oxtr* puncta counts, and higher puncta counts per cell than virgin mice (Fig. 3i–m). Fluorescence in situ HCR is a highly quantitative ISH method with single-copy detection sensitivity. The comparison with fluorescence staining in the CA3 region suggests that chromogenic in situ HCR has sensitivity and resolution comparable to fluorescence, with minimal nonspecific signal. Furthermore, the comparison between virgin and maternal mice in the MPOA suggests that the high quantitative performance is also achievable with chromogenic HCR.

A combination of DIG-conjugated hairpins and AP-conjugated anti-DIG antibodies successfully visualized *Drd2* mRNA, and neurons containing many pink–red granules were observed in the dentate gyrus polymorphic layer (DGp), while signal was not observed with the unpaired probe targeting *Drd2* and *Lrp* (Fig. 3n,o). Similarly, neurons containing many fluorescent granules were observed in the DGp in the fluorescently stained sections using SaraFluor488-labeled hairpins (Fig. 3p), and such a distribution pattern was also observed in the previous report (Etter and Krezel 2014). *Drd2*-positive puncta counts per cell were not substantially different between chromogenic and fluorescence HCR (Fig. 3q). *Drd2* expression was observed in the CP, paraventricular thalamic nucleus (PVT), endopiriform nucleus, and zona incerta (ZI) (Fig. 3r–u). These specific expression patterns are consistent with previous reports (Clark et al. 2017; Wang et al. 2019; Khlghatyan et al. 2019; Gao et al. 2020), suggesting that *Drd2* expression is faithfully visualized in chromogenic HCR.

**Fig. 4:**
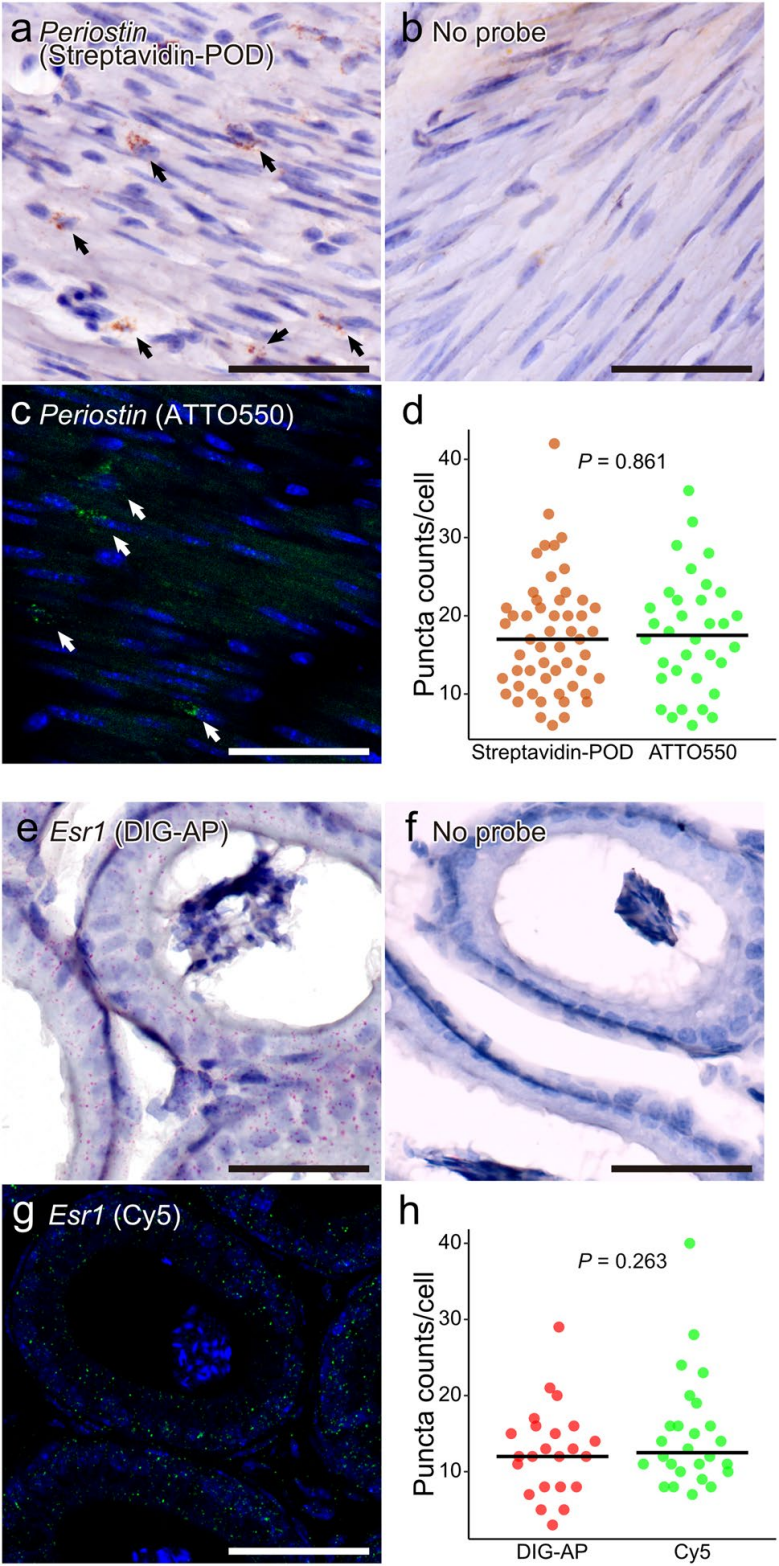
Representative images of chromogenic HCR staining for *Periostin* and *Esr1* mRNA in slide-mounted sections. **a**, **c** *Periostin* mRNA in the smooth muscle of the stomach. **a** HCR was performed using biotin-conjugated hairpin DNA, bound by POD-conjugated streptavidin, and visualized by DAB substrate. **b** No-probe control. **c** HCR was performed using ATTO550-conjugated hairpin DNA. Arrowheads indicate the *Periostin* mRNA-positive cells. **d** Comparison of puncta counts/cell between chromogenic and fluorescent HCR staining of *Periostin.* **e**, **g** *Esr1* mRNA in the epithelium of the epididymal ducts. **e** HCR was performed using DIG-conjugated hairpin DNA, bound by AP-conjugated anti-DIG antibody, and visualized by VectorRed substrate. **f** No-probe control. **g** HCR was performed using Cy5-conjugated hairpin DNA. **h** Comparison of puncta counts/cell between chromogenic and fluorescent HCR staining of *Esr1*. Scale bars: 50 μm

To assess the availability of chromogenic HCR in slide-mounted sections in addition to the floating sections, *periostin* and *Esr1* mRNA was stained by chromogenic HCR in smooth muscle of the stomach and epididymal ducts, respectively (Fig. 4). *Periostin* mRNA was visualized as brown granules using biotinylated hairpin DNA and streptavidin POD (Fig. 4a), revealing that a subset of non-spindle-shaped cells within the smooth muscle tissue were positive. These cell-type-specific signals of *periostin* mRNA were not observed in the no-probe control (Fig. 4b). The granule density in the cells and the morphology of positive cells were similar when visualized by fluorescent direct staining using Atto550-conjugated hairpin DNA (Fig. 4c,d). *Esr1* mRNA, which is known to be weakly expressed in the epididymal tubular epithelium (Zhou et al. 2002; Yamashita 2004), was detected in the epithelium of epididymal ducts using DIG-conjugated hairpin DNA and AP-conjugated anti-DIG antibodies. Many pink–red granules were observed in the ductal epithelium, whereas no signal was detected in the sperm within the lumen or in the no-probe control (Fig. 4e,f). This specific pattern was similarly observed with fluorescent direct staining using Cy5-conjugated hairpin DNA (Fig. 4g,h).

These results showed that granular signals similar to those observed with fluorescent HCR staining were also detected by chromogenic bright-field staining in both floating and mounted tissue sections. While free-floating sections allow antibody penetration from both sides, mounted sections permit access only from one side, making it difficult to achieve identical staining conditions. Nevertheless, by optimizing antibody concentration and incubation time (Table 1), we were able to obtain comparable staining intensity across free-floating and mounted sections. These observations suggest that, using the protocol proposed in this study, chromogenic detection of HCR signals can achieve sensitivity comparable to that of fluorescence-based methods. Although indirect chromogenic visualization involves more procedural steps than direct fluorescence staining, it is particularly useful for detecting mRNA in tissues with strong autofluorescence, where fluorescent signals may be obscured, and for samples intended for long-term preservation.

POD and AP each have their own advantages and limitations. POD offers superior signal localization and rapid color development, whereas AP allows for stronger signal generation through prolonged incubation in substrate-containing buffer. Although there are differences in signal contrast, the fact that similar staining results were obtained with substrates for both POD and AP indicates that the choice of enzyme and substrate combinations can be flexibly adapted to suit the target tissue and gene.

Given that fluorescent in situ HCR has been successfully applied to formalin‑fixed paraffin‑embedded (FFPE) tissues without additional procedural modifications (Wong et al. 2023; Kuboe et al. 2025), and that fluorescent and chromogenic HCR share the same underlying hybridization and amplification mechanism, we anticipate that the chromogenic HCR protocol can be directly extended to FFPE specimens. Although the present study was performed on cryosections, preliminary experiments indicate that this chromogenic HCR protocol is applicable to FFPE tissues. Future work will include quantitative validation of chromogenic HCR performance in clinical FFPE material.

### Duplex chromogenic HCR staining

**Fig. 5:**
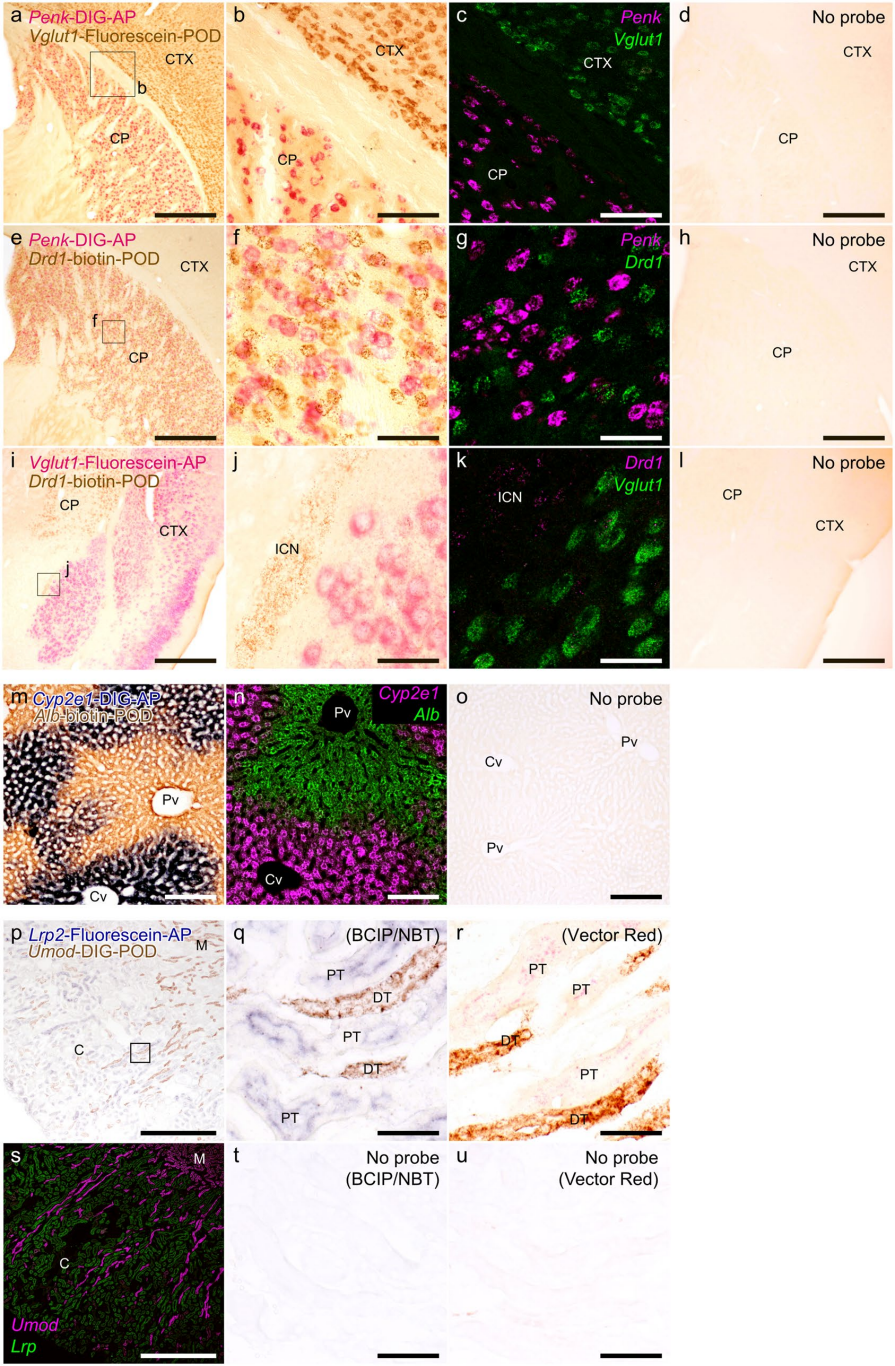
Representative images of duplex chromogenic HCR staining and corresponding fluorescent HCR staining. **a**–**l** Duplex staining in the mouse brain. **a**, **b** *Penk* and *Vglut1* mRNA was detected using a combination of DIG/AP-labeled anti-DIG antibody with VectorRed and fluorescein/POD-labeled anti-fluorescein antibody with DAB, respectively. **c** *Penk* and *Vglut1* fluorescent HCR staining of the same fields. **d** No-probe control. **e**, **f** *Penk* and *Drd1* mRNA was detected using a combination of DIG/AP-labeled anti-DIG antibody with VectorRed and biotin/POD-labeled streptavidin with DAB, respectively. **g** *Penk* and *Drd1* fluorescent HCR staining of the same fields. **h** No-probe control. **i**, **j** *Vglut1* and *Drd1* mRNA was detected using a combination of fluorescein/AP-labeled anti-fluorescein antibody with VectorRed and biotin/POD-labeled streptavidin antibody with DAB, respectively. **k** *Vglut1* and *Drd1* fluorescent HCR staining of the same fields. **h** No-probe control. Panels **b**, **f**, **j** show magnified views of the rectangle regions indicated in (**a**, **e**, **i**). **m** *Cyp2e1* and *albumin* mRNA in the mouse liver was detected using a combination of DIG/AP-labeled anti-DIG antibody with BCIP/NBT and biotin/POD-labeled streptavidin with DAB, respectively. **n** Fluorescent HCR staining of the same region as (**m**). **o** No-probe control. **p** *Lrp2* and *Umod* mRNA in the mouse kidney was detected using a combination of fluorescein/AP-labeled anti-fluorescein antibody with BCIP/NBT and biotin/POD-labeled streptavidin with DAB, respectively. **s** Fluorescent HCR staining of the same region as (**p**). **q** Magnified view of the rectangle region indicated in (**p**). **r** Renal cortex image from the sections stained using VectorRed as the substrate for AP instead of BCIP/NBT. **t**, **u** No-probe control. Scale bars: 500 μm (**a**, **c**, **d**, **e**, **g**, **h**, **i**, **k**, **l**, **p**, **s**), 100 μm (**b**, **f**, **j**), 200 μm (**m**–**o**), 25 μm (**q**, **r**, **t**, **u**). *CP* caudate putamen, *CTX* cortex, *ICN* intercalated nucleus, *Pv* portal vein, *Cv* central vein, *C* cortex, *M* medulla, *PT* proximal tubule, *DT* distal tubule

To evaluate feasibility of simultaneous visualization of two target mRNAs, probe hybridization and HCR reactions for two targets were performed simultaneously, followed by sequential hapten detection and chromogenic staining using the same procedure as for the single staining (Fig. 5). In mouse brain, *Penk* mRNA is specifically expressed in the subset of neurons within the CP, where the somata exhibited intense red staining using AP and VectorRed substrate. *Vglut1* mRNA expression is specific to the cerebral cortex, with cells showing intense red and brown signals upon POD/DAB and AP/VectorRed staining, respectively (Fig. 5a,b and i,j). *Drd1* is expressed in a distinct neuronal subset from *Penk* mRNA-positive neurons in the CP (Gokce et al. 2016), and brown-stained granules of *Drd1* by POD/DAB were also observed in the intercalated cell nucleus (ICN; Fig. 5j). These red and brown signals were separable and maintained specificity in expression patterns, even under conditions of duplex staining, and the direct fluorescent staining using fluorophore-conjugated hairpin DNA showed comparable distributions (Fig. 5c,g,k). Such specific staining was not observed in the no-probe control (Fig. 5d,h,l). Importantly, the comparable signal intensity observed under dual-staining conditions indicates that chromogenic HCR retains its sensitivity and can be applied with the same reliability as in single-staining setups. This expands its utility in multiplexed histological analyses without compromising detection performance.

In the liver, there is a gene expression gradient from the portal to central venous regions, and *albumin* and *Cyp2e1* mRNA is complementarily expressed along this axis (Ghafoory et al. 2013; Hu et al. 2022). *Albumin* mRNA was labeled with biotinylated hairpin DNA and visualized chromogenically using streptavidin POD and DAB, showing strong expression in the hepatocyte around the portal vein (Fig. 5m). In parallel, *Cyp2e1* mRNA was labeled with DIG-conjugated hairpin DNA and visualized using AP-conjugated anti-DIG antibody and BCIP/NBT substrate. *Cyp2e1* mRNA was strongly and specifically expressed in hepatocytes around the central vein. These trends were also confirmed in the fluorescent direct staining (Fig. 5n), and no-probe control experiments showed endogenous biotin or enzyme did not produce the region-specific staining (Fig. 5o). These findings suggest that dual staining can be successfully performed not only in floating sections but also in mounted sections, with chromogenic HCR yielding results comparable to fluorescent staining. However, in cases involving highly abundant mRNA such as *albumin* or *Cyp2e1* in the liver, the nature of bright-field detection makes it challenging to determine whether two transcripts are colocalized within the same cell. This limitation warrants caution and indicates that such applications may not be practical. Therefore, to reliably assess colocalization in dual staining setups, careful selection and optimization of enzyme–substrate combinations are essential. It should be noted that dark counterstaining may interfere with the visualization of mRNA signals, and thus requires careful optimization to avoid masking target-specific staining.

In the kidney, *Lrp2* and *Umod* were expressed as markers of proximal and distal tubules, respectively (Tokonami et al. 2018; Rudman-Melnick et al. 2020; Aceves et al. 2022). *Lrp2* mRNA was labeled with fluorescein-conjugated hairpin DNA and visualized using AP-conjugated anti-fluorescein antibody with either BCIP/NBT or VectorRed as the AP substrate. *Umod* mRNA was labeled with DIG-conjugated hairpin DNA and visualized using POD-conjugated anti-DIG antibody and DAB. The kidney contains abundant endogenous biotin, and our preliminary no-probe control experiments showed strong background signals attributable to endogenous biotin. Therefore, we avoided biotin–streptavidin-based detection in this tissue and instead used DIG- and fluorescein‑based detection systems. As expected, the expression of *Lrp2* and *Umod* showed exclusive distribution. *Lrp2* mRNA was confined to tubes in the renal cortex, while *Umod* mRNA was observed both in the cortex and medulla, suggesting specific staining of the proximal and distal tubules, respectively (Fig. 5p,q,r). These staining patterns were consistent with those obtained by fluorescent direct staining (Fig. 5s). To compare two major AP substrates for the chromogenic visualization, epithelial cells in the proximal tubules were stained blue and red when BCIP/NBT and VectorRed were used, respectively. No signal was detected in either negative control using BCIP/NBT or VectorRed as the chromogenic substrate (Fig. 5t,u). The overall staining distribution was similar between these two AP substrates used for *Lrp2* mRNA detection, and the signals were biased toward the luminal side and located beneath the apical surface of the proximal tubules. The staining characteristics varied depending on the substrate used. BCIP/NBT produced diffuse cytoplasmic staining, whereas VectorRed yielded concentrated, granular signals (Fig. 5q,r). In addition to their distinct optical properties, which directly affect microscopic visualization, the precision of pigment localization and the stability of the reaction product are also critical factors influencing the overall staining outcome. BCIP/NBT, which produces a blue-to-purple precipitate, offers excellent contrast and visibility against the brown coloration of DAB. However, owing to its tendency to diffuse, it may be less suitable for precise localization of mRNA signals. In bright-field microscopy, spatial resolution is inherently limited by light diffraction and the spread of chromogenic precipitates, resulting in lower resolution than fluorescence-based detection. Bright-field imaging also lacks the optical sectioning capability of confocal microscopy, making it difficult to resolve signals from overlapping cells along the *z*-axis. Consequently, signals from adjacent cells or closely spaced subcellular compartments can appear to overlap, even when mRNA is expressed in distinct cells or regions. For these reasons, true colocalization at the single-cell level cannot be reliably assessed by bright-field chromogenic duplex staining alone. Although duplex chromogenic HCR can reveal regional co-expression patterns, it does not provide sufficient spatial resolution to unequivocally determine co-expression within individual cells. In tissues with high cell density or overlapping signals, apparent color mixing may reflect signal overlap from adjacent cells rather than true co-expression. However, when a brightly stained, highly expressed cellular marker is combined with a punctate mRNA signal stained in a darker color, colocalization can sometimes be inferred at the single-cell level, provided the signals are clearly distinct and non-overlapping. For rigorous assessment of single-cell co-expression, complementary approaches such as fluorescent duplex HCR or single-cell reverse transcription polymerase chain reaction (PCR) should be considered.

**Fig. 6:**
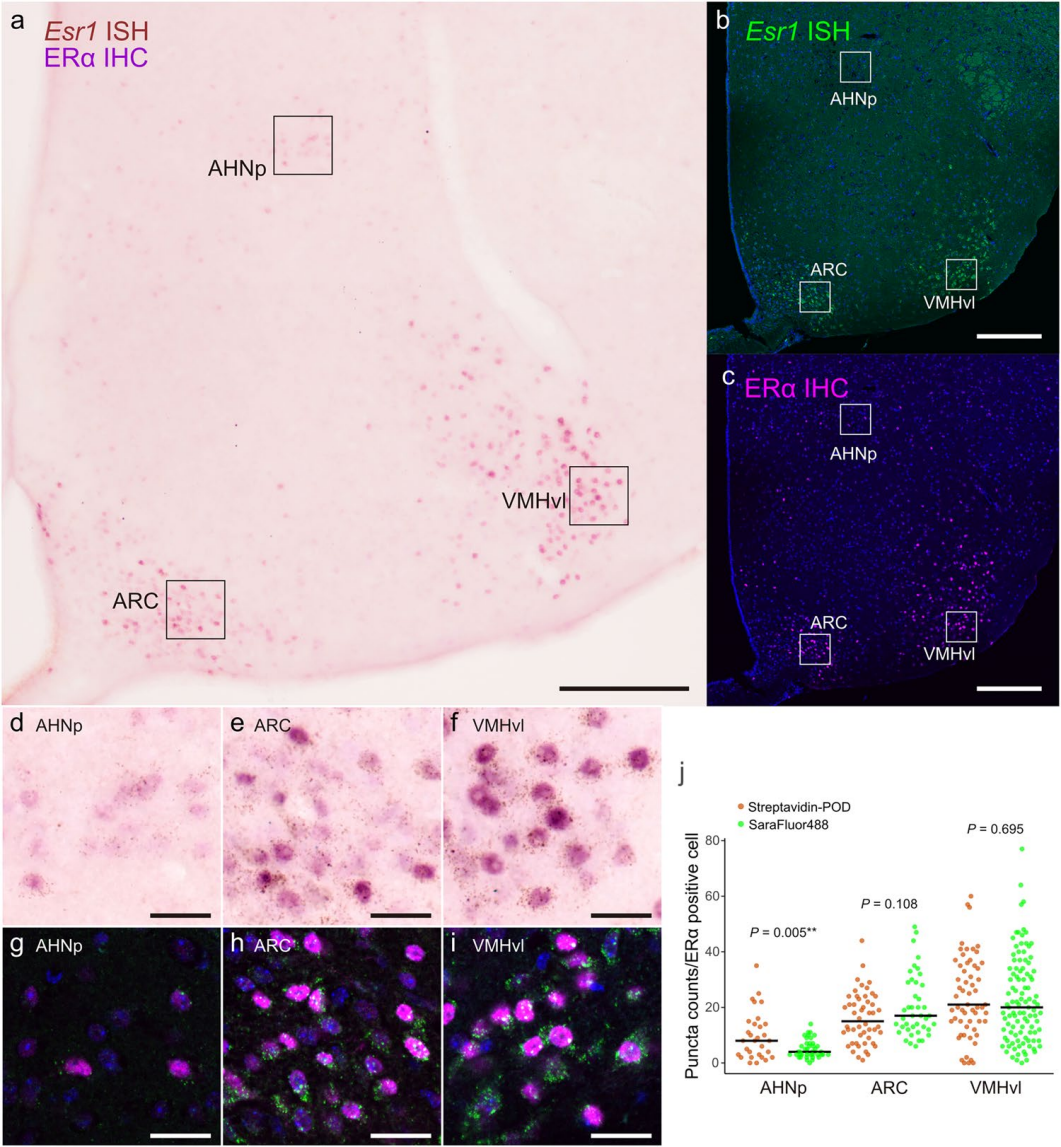
Representative images of in situ HCR staining for *Esr1* mRNA and immunostaining for ERα protein in the mouse hypothalamus. **a**–**c** Wide-field view of HCR staining sections. **a**, **d**–**f** *Esr1* mRNA was visualized using a combination of biotin-conjugated hairpin DNA, POD-conjugated streptavidin, and DAB substrate. **b**, **c**, **g**–**i** Fluorescent staining of *Esr1* mRNA and ERα protein. **d**–**i** Magnified view of regions marked by rectangles (**a**–**c**). **j** Comparison of puncta counts/ERα-positive cell between chromogenic and fluorescent HCR staining of *Esr1*. Scale bars: 200 μm (**a**–**c**) or 25 μm (**d**–**i**). *AHNp* anterior hypothalamic nucleus, posterior part, *ARC* arcuate nucleus, *VMHvl* ventromedial hypothalamic nucleus, ventrolateral part

Next, *Esr1* mRNA and its encoded protein, estrogen receptor alpha (ERα), were visualized in the mouse hypothalamus using a combination of in situ HCR and immunostaining (Fig. 6). In mouse hypothalamus, *Esr1* mRNA is known to be highly expressed in the arcuate nucleus (ARC), ventrolateral part of the ventromedial hypothalamus (VMHvl), and anterior hypothalamic nucleus pars posterior (AHNp) (Simerly et al. 1990; Shughrue et al. 1997; Mitra et al. 2003). Using chromogenic and fluorescent staining, we confirmed the presence of both granule-like signals of *Esr1* mRNA and nuclear ERα immunoreactivity in the ARC, VMHvl, and AHN (Fig. 6a–c). The number of *Esr1*-positive granules per cell was high in the ARC and VMHvl, whereas the AHNp exhibited a lower density of *Esr1* positive granules per cell (Fig. 6d–i). Similarly, the ERα immunoreactivity was more intense in the ARCs and VMHvl and weaker in the AHNp (Fig. 6d–i). However, when the positive cells were observed separately, the number of *Esr1* mRNA-positive granules and intensity of ERα immunostaining did not always correlate. In some cells, a high number of *Esr1*-positive granules was accompanied by weak ERα immunoreactivity. These patterns were consistently observed in both chromogenic and fluorescent staining (Fig. 6d–i). These staining results reinforce a key advantage of in situ HCR using short hairpins, namely its compatibility with costaining by immunohistochemistry (Tsuneoka and Funato 2020). In the ARC and VMH, the number of Esr1 mRNA puncta per ERα-positive cell did not differ between chromogenic and fluorescence HCR, whereas a significant difference was observed in the AHNp (Fig. 6j). The difference in the AHNp may be due to regional variations in background signal, particularly in fluorescence observations, where part of the signals may have fallen below the detection threshold. Chromogenic HCR staining delivered sensitivity and spatial resolution comparable to fluorescent HCR when combined with IHC, supporting its use as an effective alternative for experiments requiring simultaneous detection of IHC. The combination of nuclear IHC markers and cytoplasmic HCR granules is especially useful for assessing intracellular colocalization because the nuclear signal supplies a clear cellular landmark, while HCR reveals transcript distribution, reducing ambiguity when assigning signals to the same cell. In many ISH methods, treatments such as pepsin or proteinase K digestion are required to enhance probe penetration and ensure sensitivity (Tsuneoka and Funato 2020). However, these treatments often markedly reduce the sensitivity of subsequent immunostaining and add complexity by increasing the number of procedural steps. In contrast, the in situ HCR method employing short hairpins enables chromogenic staining through a streamlined protocol and offers the advantage of allowing immunostaining to be performed sequentially.

The relationship between *Esr1* mRNA expression and ERα protein abundance is notable. The regional trends of expression level of mRNA were similar to those of protein. This parallel pattern supports region-specific regulation of *Esr1* transcription and indicates that the in situ HCR accurately identifies the subpopulation of *Esr1* expressing cells in the hypothalamus. However, discordance between mRNA and protein levels in individual cells suggests that either transcription, translation, or stability of mRNA and protein differs cell by cell, or that the peaks of mRNA and protein expression are temporally offset (Liu et al. 2016; Genshaft et al. 2016).

Our chromogenic HCR protocol offers several practical advantages compared with established chromogenic ISH platforms such as RNAscope, a branched DNA-based assay. First, regarding workflow complexity, chromogenic HCR does not require protease K digestion under the conditions used here, whereas RNAscope-type assays typically rely on protease treatment. Second, regarding cost, the reagent cost per sample for chromogenic HCR is substantially lower than that of commercial RNAscope assays; in our experience in Japan, the cost is less than one-tenth of that for RNAscope. Third, with respect to instrumentation, chromogenic HCR can be performed using standard laboratory equipment without specialized devices. In terms of multiplexing, chromogenic HCR is limited to two colors in bright-field microscopy owing to the availability of chromogenic substrates and the difficulty of distinguishing more than two colors. Advantages of chromogenic HCR include its simple workflow, low cost, no requirement for specialized equipment, and flexibility in probe design. Limitations include restricted multiplexing capacity and the need to optimize antibody/streptavidin-based detection conditions for each tissue type, whereas RNAscope-type assays do not rely on antibodies for signal detection. Despite these limitations, chromogenic HCR provides a cost-effective and accessible alternative to commercial branched DNA platforms, particularly for research laboratories seeking flexibility in probe design and simplified workflows.

In conclusion, we confirmed, across various specimen types, that the chromogenic staining method we developed using in situ HCR achieves detection sensitivity comparable to that of in situ HCR fluorescence staining. Importantly, in situ HCR allows staining to be performed with the same protocol across multiple platforms, and even in bright-field chromogenic staining applications, the HCR reaction can be carried out using the identical protocol. Since differences in haptens, detection systems, and chromogenic substrates can substantially affect staining behavior, optimization of these components will be necessary. Nevertheless, this approach enables double staining without compromising the compatibility with immunostaining, thereby expanding the options for specimens in which fluorescence staining is difficult to apply.

## Supplementary Information

Below is the link to the electronic supplementary material.

Supplementary file1 (DOCX 39 KB)

## Data Availability

The datasets generated during and/or analyzed during the current study are available from the corresponding author on reasonable request.
