# Supplementary material for: Methodological framework for chromogenic mRNA detection using in situ hybridization chain reaction
Source: Histochem Cell Biol. 2026 May 6;164(1):32. doi: 10.1007/s00418-026-02482-w (PMC13149615; doi:10.1007/s00418-026-02482-w)
Supplement: Supplementary file 1 — Supplementary file1 (DOCX 39 KB) [file 418_2026_2482_MOESM1_ESM.docx]

**Supplementary information**

**“Methodological Framework for Chromogenic mRNA Detection Using *in situ* Hybridization Chain Reaction”**

**in Histochemistry and Cell Biology**

Mitsuru Yashiro^1, 2^ (0000-0002-6147-9153), Yousuke Tsuneoka^1^* (0000-0002-1028-054X), Yusuke Atsumi^1, 2^, Aki Makanae^1, 2^, Hiromasa Funato^1, 3^* (0000-0002-2787-9700).

1) Department of Anatomy, Faculty of Medicine, Toho University, Tokyo, Japan.

2) Center for Research and Product Development, Nepa Gene Co., Ltd., Chiba, Japan.

3) International Institutes for Integrative Sleep Medicine (WPI-IIIS), University of Tsukuba, Ibaraki, Japan.

*Corresponding Authors

E-mail: yousuke.tsuneoka@med.toho-u.ac.jp; hiromasa.funato@med.toho-u.ac.jp

Telephone: +81-3-3762-4151

Table S1. Split-initiator probe sequences

| Target mRNA  and probe name | First probe | Second probe |
| --- | --- | --- |
| Albumin |  |  |
| Albumin-1S45 | CCTCCACGTaaTGTGTGCTTCTCGGCGAAACACACC | CATTATACCGATGGGCGATCTCACTaaTCCATCTAAGCT |
| Albumin-2S45 | CCTCCACGTaaCTGTTACTTCCTGCACTAATTTGGC | CATCGGCAACACACGTCTTTGCAAAaaTCCATCTAAGCT |
| Albumin-3S45 | CCTCCACGTaaTTGGAATGGCACACAACTTATCTCC | CCAGTTCACCATAGTTTTCACGGAGaaTCCATCTAAGCT |
| Albumin-4S45 | CCTCCACGTaaCATTGTACTGCTCAGCATAGTAAAG | CCTCTGCACAACACTGGGTCAGAATaaTCCATCTAAGCT |
| Albumin-5S45 | CCTCCACGTaaACGGACAGATGAGACCAATGCTTTC | CTGCATACTGGAGCACTTCATTCTCaaTCCATCTAAGCT |
| Albumin-6S45 | CCTCCACGTaaAGACGAGCTACTGCCCATGCTTTAA | AAGTCAGCATTGGGGAATGTCTGGCaaTCCATCTAAGCT |
| Albumin-7S45 | CCTCCACGTaaGTCAGGTCTGTTGCCAATTTGGTGA | CCATGGCAGCACTCCTTGTTGACTTaaTCCATCTAAGCT |
| Albumin-8S45 | CCTCCACGTaaAGTTTGCTGGAGATAGTCGCCTGGT | AACAGTGGTTTATCGCAGCAAGTCTaaTCCATCTAAGCT |
| Albumin-9S45 | CCTCCACGTaaTCATGCTCCACCTCACTAAGACAGT | ATGGCAGGCAGATCAGCAGGCATGGaaTCCATCTAAGCT |
| Albumin-10S45 | CCTCCACGTaaCCGTAGCATGCGGGAGGATTGGCTT | AGAGGCTGAAATTCAGCAAGCACTGaaTCCATCTAAGCT |
| Albumin-11S45 | CCTCCACGTaaCTAGAATGGCATTTTGGAATCCATA | CCTGAGGTGCTTTCTGGGTGTAGCGaaTCCATCTAAGCT |
| Albumin-12S45 | CCTCCACGTaaTTCTTGCAGCCTCCACGAGAGTTGG | AACACTTGGTGCCCACTCTTCCTAGaaTCCATCTAAGCT |
| Albumin-13S45 | CCTCCACGTaaCACAAGGCAGTCTCTGATCTTCAGG | TCAGGATTGCAGACAGATAGTCTTCaaTCCATCTAAGCT |
| Albumin-14S45 | CCTCCACGTaaCTGGGGTCTTCTCATGCAGCAGACA | TACAGCACTTGGTAACATGCTCACTaaTCCATCTAAGCT |
| Albumin-15S45 | CCTCCACGTaaAGGTCTCAGCTTTAAACTCTTTGGG | GTGTGCAGATATCAGAGTGGAAGGTaaTCCATCTAAGCT |
| Albumin-16S45 | CCTCCACGTaaAGCCGTTTGTTTCTTAATCTGCTTC | GGGCTTGTGCTTCACCAGCTCAGCAaaTCCATCTAAGCT |
| Albumin-17S45 | CCTCCACGTaaCATGACAGTCTTCAGTTGCTCCGCT | TGTATCCAGGAACTGTGCAAAGTCAaaTCCATCTAAGCT |
| Albumin-18S45 | CCTCCACGTaaAGAAGCAGGTGTCCTTGTCAGCAGC | TAGTGACAAGGTTTGGACCCTCAGTaaTCCATCTAAGCT |
|  |  |  |
| Cytochrome P450 2E1 |  |  |
| Cyp2e1-1S72 | CGGTGGAGTaaCCTTGTAGCCATGCAGGACCACGAT | TGTGGTTCAGTAGCACCTCCTTGACaaGGCAAGTAGAT |
| Cyp2e1-2S72 | CGGTGGAGTaaCCTGGAACACAGGAATGTCCCCTCG | TGAAAATAATCCCCTTGTTCTTGTAaaGGCAAGTAGGAT |
| Cyp2e1-3S72 | CGGTGGAGTaaAAAACCTCCGCACGTCCTTCCATGT | TTCCCCAGTCACGGAGGATACTTAGaaGGCAAGTAGGAT |
| Cyp2e1-4S72 | CGGTGGAGTaaGTCAAAAGGCTGGCCTTTGGTCTTT | GGGTGCACAGCCAATCAGAAAGGTAaaGGCAAGTAGGAT |
| Cyp2e1-5S72 | CGGTGGAGTaaTTCATTGAACAAACTCATGAGCTCC | CCAGGGAGTACTCAGCAGGTAGAAGaaGGCAAGTAGGAT |
| Cyp2e1-6S72 | CGGTGGAGTaaTTCCTTGGCTTTTCCAAGTGTGTAC | GCAGTTGATGTCCAGTGACTTAAGGaaGGCAAGTAGGAT |
| Cyp2e1-7S72 | CGGTGGAGTaaTCTCTATGAGGAGACAGTCAGTCAC | GTTCTTGGCTGTGTTTTTCCTTCTCaaGGCAAGTAGGAT |
| Cyp2e1-8S72 | CGGTGGAGTaaGTAGGGCATATTCATCCTGTCTCGG | CTGAATCTCATGCACTACAGCGTCCaaGGCAAGTAGGAT |
| Cyp2e1-9S72 | CGGTGGAGTaaCGGAACACGGTGTCTCGGGTTGCTT | ACTGTACCCTTGGGGATGACATATCaaGGCAAGTAGGAT |
| Cyp2e1-10S72 | CGGTGGAGTaaATAGTCACTGTACTTGAACTTCCCA | GCGCTTTCCTGCAGAAAACGCCTTGaaGGCAAGTAGGAT |
| Cyp2e1-11S72 | CGGTGGAGTaaCAGAGACTTCAGATTAAAATGCTGC | GCTGAGGTCGATATCCTTAGGGTCAaaGGCAAGTAGGAT |
| Cyp2e1-12S72 | CGGTGGAGTaaTCGCGTGGGATACTGCCAAAGCCAA | GAACGAGGAATGACACAGAGTTTAAaaGGCAAGTAGGAT |
|  |  |  |
| Dopamine receptor D1 |  |  |
| Drd1-1S41 | GCTCGACGTaaAGCCTAAAATACATGCATTTCTCCT | GGGATGCTGCCTCTTCTTCTGAGACaaTCCTTTGCAACA |
| Drd1-2S41 | GCTCGACGTaaCACCTGTCTTCTGGGTTCAGTGCTC | TACCAGGAAGAAGAGCCGCTTGCTTaaTCCTTTGCAACA |
| Drd1-3S41 | GCTCGACGTaaCGGTGGCTTAGCCCTCACGTTCTTG | AGCCATCTTCCAGAAGGAAAGCACTaaTCCTTTGCAACA |
| Drd1-4S41 | GCTCGACGTaaAACAGGCTGTGAGGATGCGAAAGGA | GAGTGGACAGGATAAGCAGGGACAGaaTCCTTTGCAACA |
| Drd1-5S41 | GCTCGACGTaaACAGTCCTTGGAGATGGAGCCTCGG | AGCATGAGGGATCAGGTAAACCAGAaaTCCTTTGCAACA |
| Drd1-6S41 | GCTCGACGTaaCCGCTGTGGGTAACGGGTTGGATCT | GAACCCAATATTCAGGTTGAATGCTaaTCCTTTGCAACA |
| Drd1-7S41 | GCTCGACGTaaGTTGGAGAAGTTCTGTAACTGTCCA | GGCTAGAGAATACACTGGAGTCAGTaaTCCTTTGCAACA |
| Drd1-8S41 | GCTCGACGTaaGAATCTCAGAGTCTATGTGTACTGT | GCCTTCCCAGAAGTCATTCCCAGCTaaTCCTTTGCAACA |
| Drd1-9S41 | GCTCGACGTaaCATTCACCTCGATGCACATCTTAAT | GCTGCTGTGACCTGTGACTTTACAGaaTCCTTTGCAACA |
| Drd1-10S41 | GCTCGACGTaaCGATGAGGCACAGCTCATTAGCTAG | CACCAACAACAGAAGTGTATACTTCaaTCCTTTGCAACA |
| Drd1-11S41 | GCTCGACGTaaCTAGATTCAAAACCACAGGGAGGAA | TTTGCAGTCCAGGCTATGACTTGGAaaTCCTTTGCAACA |
| Drd1-12S41 | GCTCGACGTaaCAGCTGCTTCCAAAGAACACCTTGT | CCTTCCTTCAGTTCTATCCACATGTaaTCCTTTGCAACA |
| Drd1-13S41 | GCTCGACGTaaTATAGCATCCTAAGAGGGTCGAGAA | TGGCATTATTCGTTGTAGGGCAGAGaaTCCTTTGCAACA |
| Drd1-14S41 | GCTCGACGTaaTCTCACACTTCCTTCCTGGAAAGTT | AGCACCTGTTTGGATACAGCAAACAaaTCCTTTGCAACA |
| Drd1-15S41 | GCTCGACGTaaCCAGCGATGAGCCCAACTATCGGAG | CTTGGTTAGACCTGGGCAGATGAAGaaTCCTTTGCAACA |
| Drd1-16S41 | GCTCGACGTaaTGATGCTCAACACACACCGGGAAGG | TTGTGACTAGTGATGCTGATGGCTTaaTCCTTTGCAACA |
| Drd1-17S41 | GCTCGACGTaaCCACCGGCCTCCTCCCTCTTCAGGT | GACAGCTTCTCCAGTGGCTTAGGTAaaTCCTTTGCAACA |
| Drd1-18S41 | GCTCGACGTaaAGCCCTAGGGAACTCATAGCTTCAG | GGGTCTCGTGTTAATTTCTTAACAGaaTCCTTTGCAACA |
| Drd1-19S41 | GCTCGACGTaaCAGTTCAGTCTCTGATTTAACATTG | TGGCTCATGAAGTGCTAGTACAATGaaTCCTTTGCAACA |
| Drd1-20S41 | GCTCGACGTaaTTGAGTTAAGGAACCACCACATCAG | CTTACCTTGTGTTTATTTGTGTCTGaaTCCTTTGCAACA |
|  |  |  |
| Dopamine receptor D2 |  |  |
| Drd2-1P1S23 | GGGTGGTCGaaAGGACAGGTTCAGTGGATCCATTGG | TCTGCCTCTCCAGATCATCATCGTAaaTCGAAGTCGTAT |
| Drd2-2P1S23 | GGGTGGTCGaaTTGCCCTTGAGTGGTGTCTTCAGGT | AGTTTCATGTCCTCAGGGTGGGTACaaTCGAAGTCGTAT |
| Drd2-3P1S23 | GGGTGGTCGaaGCTATGTAGACCGTGGTGGGATGGA | TTCTGGTTTGGCAGGACTGTCAGGGaaTCGAAGTCGTAT |
| Drd2-4P1S23 | GGGTGGTCGaaTCCTGCGGCTCATCGTCTTAAGGGA | CTTTCTTCTCCTTCTGCTGGGAGAGaaTCGAAGTCGTAT |
| Drd2-5P1S23 | GGGTGGTCGaaTCAGGATGTGCGTGATGAAGAAGGG | GTGGGATGTTGCAGTCACAGTGTATaaTCGAAGTCGTAT |
| Drd2-6P1S23 | GGGTGGTCGaaTGTTGAAGGTGGTATAGATGATGGG | TCTTCATGAAGGCCTTGCGGAACTCaaTCGAAGTCGTAT |
| Drd2-7P1S23 | GGGTGGTCGaaGGAGACAGGATCTGCATGTGAAAGG | GGAAGGCTGCTGCTTCTTTGGTGCCaaTCGAAGTCGTAT |
| Drd2-8P1S23 | GGGTGGTCGaaCAAGGGCCAGGCCAAGCCAACAATC | TCTACCACCTGATCCAACATAGGCAaaTCGAAGTCGTAT |
| Drd2-9P1S23 | GGGTGGTCGaaCACTAACGTCACCTGGAGGCCTTGG | TACCTGGTGTTTATGTGTGTCTCCCaaTCGAAGTCGTAT |
| Drd2-10P1S23 | GGGTGGTCGaaTGCTATGGAAAGTAGGGATTCACAC | AACAGTGGAGAACATAGCAATACCCaaTCGAAGTCGTAT |
| Drd2-11P1S23 | GGGTGGTCGaaGTGGATTGGAGGGTAGAGGGAAATC | GTCCAATGTATGTCAAGAAGTGGCGaaTCGAAGTCGTAT |
| Drd2-12P1S23 | GGGTGGTCGaaCCCATTAGACTTCATGATAACGGTG | CATTCTCCGCCTGTTCACTGGGAAAaaTCGAAGTCGTAT |
| Drd2-13P1S23 | GGGTGGTCGaaGATTGACAATCTTGGCATGCCCATT | GGATCTCAAAGAACTTGGCAATCCTaaTCGAAGTCGTAT |
| Drd2-14P1S23 | GGGTGGTCGaaGAAGGCCGAGTCCATCTGGGCCTTT | AACACTGCAGAGCCTGCAGGGTCAAaaTCGAAGTCGTAT |
| Drd2-15P1S23 | GGGTGGTCGaaGCCCTACCCTGGCAGGATAAGGGTG | AGAGCTGGTAAGATACCAGTCTCCCaaTCGAAGTCGTAT |
| Drd2-16P1S23 | GGGTGGTCGaaAACTTCCCAGGCTGTCCAGCATCTG | TTCAGCTCCAACACTGGTCCAAGGCaaTCGAAGTCGTAT |
| Drd2-17P1S23 | GGGTGGTCGaaGGGGAGAGGGAAGATATCAACACCC | GCAGAGGCACTGGCAGAGAAGAGACaaTCGAAGTCGTAT |
| Drd2-18P1S23 | GGGTGGTCGaaTGTTACAGAGTTGGAGCCCAGACCC | TTTATTGGTTTGGTGCATGTATGGTaaTCGAAGTCGTAT |
| Drd2-19P1S23 | GGGTGGTCGaaCCTGTCTGGCTTCCCTTCGGACCCA | CAGCATGGCATAGTAGTTGTAGTGGaaTCGAAGTCGTAT |
| Drd2-20P1S23 | GGGTGGTCGaaACGCTTGCGGAGAACGATGTAGATT | GCTACGCTTGGTGTTGACCCGCTTCaaTCGAAGTCGTAT |
| Drd2-21P1S23 | GGGTGGTCGaaAGCTCCTGAGCTCGGCGGGCAGCAT | CTGGTGCTTGACAGCATCTCCATTTaaTCGAAGTCGTAT |
|  |  |  |
| Estrogen receptor 1 |  |  |
| Esr1-1A161 | GGTACGCGAaaGTAGAATCCGTTCCGAGGCTGTTAT | GCAGGGAGCCTTGTGTTTTCGGTTTaaAGGTAGGTGTAA |
| Esr1-2A161 | GGTACGCGAaaCTTAAGCTAGACAATGGCAGAGGAG | AGAAGAATGTCGCCCAGAGACTGCCaaAGGTAGGTGTAA |
| Esr1-3A161 | GGTACGCGAaaAGGGTCATGGTCATGGTAAGTGGCA | AAGGCCATTCCCGAGGCTTTGGTGTaaAGGTAGGTGTAA |
| Esr1-4A161 | GGTACGCGAaaTTGTCAGAATTAGACCTGTAGAAGG | AGTCTCTCTCGGCCATTCTGGCGTCaaAGGTAGGTGTAA |
| Esr1-5A161 | GGTACGCGAaaCTTGTGCTTCAACATTCTCCCTCCT | TCGGCCTTCCAAGTCATCTCTCTGAaaAGGTAGGTGTAA |
| Esr1-6A161 | GGTACGCGAaaTGTCTCCTGAAGCACCCATTTCATT | GGCTTGGCCAAAGGTTGGCAGCCCTaaAGGTAGGTGTAA |
| Esr1-7A161 | GGTACGCGAaaCAGGGCTATTCTTCTTAGTGTGCTT | CCATCTGGTCAGCTGTCAAGGACAAaaAGGTAGGTGTAA |
| Esr1-8A161 | GGTACGCGAaaTAAGCCCATCATTGAGGCTTCACTG | CAGCTCCCTATCTGCTAGGTTGGTCaaAGGTAGGTGTAA |
| Esr1-9A161 | GGTACGCGAaaCTCTCTTTGCCCAGTTGATCATATG | GGAGATTCAAGTCCCCAAAGCCTGGaaAGGTAGGTGTAA |
| Esr1-10A161 | GGTACGCGAaaCACTCGAGAAGGTGGACCTGATCAT | CCAATCATCAGAATCTCCAGCCAGGaaAGGTAGGTGTAA |
| Esr1-11A161 | GGTACGCGAaaTGGAGCTGGTGGTGGCCAGCTGGGT | AGTAGGTTTGTAAGGAATGTGCTGAaaAGGTAGGTGTAA |
| Esr1-12A161 | GGTACGCGAaaGAGCTCTCAGATCGTGTTGGGGAAG | ATTCTCAGAACCTTTCGGGGAGCCTaaAGGTAGGTGTAA |
| Esr1-13A161 | GGTACGCGAaaGAGACAGAATTCTGCTAGTCATACA | GGTGGATGCATGCCACAGTGTACGCaaAGGTAGGTGTAA |
| Esr1-14A161 | GGTACGCGAaaAACAGAAGACATGCCACTAAGAACT | GCCCTGGAATCCCTTTTGCCTGTTCaaAGGTAGGTGTAA |
| Esr1-15A161 | GGTACGCGAaaGCCCCAGGGGCAGAAGACTATTATA | CAGGTCCTCAAGCTGCCTTTACTGCaaAGGTAGGTGTAA |
| Esr1-16A161 | GGTACGCGAaaTGCTGAAGAATGTCCAGTTTACCAG | CAGGGAAGAGACAAGGCTAGCATCCaaAGGTAGGTGTAA |
| Esr1-17A161 | GGTACGCGAaaTTACTTTCCTTCTTAGACTCCTCCG | GGCCAGGTCATTCTCCACATTTCTCaaAGGTAGGTGTAA |
| Esr1-18A161 | GGTACGCGAaaCCAGAGCAGCCTCACTGTGCATGGT | AATGTAAGTGAAGTCAGCCCATGGAaaAGGTAGGTGTAA |
| Esr1-19A161 | GGTACGCGAaaACAGGTATCTGATGTCACCTCTTCC | CCAAGTTCCTGGAGCATCTACAGGAaaAGGTAGGTGTAA |
| Esr1-20A161 | GGTACGCGAaaTTGGGTGGCTGTGGATAGAGTAAGT | ACTAGAGAGACCAAGACCCCAGAGGaaAGGTAGGTGTAA |
|  |  |  |
| LDL receptorrelated protein 2 |  |  |
| Lrp2-1S23 | GGGTGGTCGaaAGAGGGTTTGGCAAGGCTGCTTAAG | TCTGAAAGTCACCCCGTTTCCATTTaaTCGAAGTCGTAT |
| Lrp2-2S23 | GGGTGGTCGaaTGTCCATGTTCACATCAGCCCCTGA | TCTCAGGGCCAAAAGGAGACACACCaaTCGAAGTCGTAT |
| Lrp2-3S23 | GGGTGGTCGaaATTGCCATGGATCTGTCAATAATTG | CCTACCTCCATGACGAATTGTTCATaaTCGAAGTCGTAT |
| Lrp2-4S23 | GGGTGGTCGaaGTTTTCAAATATCACAGGCTGCTTG | AGTACTGTCTTTGGCTGCATACATTaaTCGAAGTCGTAT |
| Lrp2-5S23 | GGGTGGTCGaaCCTGCACTGCCAGGCCCACTTTGGA | CAGTCACCTGTGAGCTTACTGACGGaaTCGAAGTCGTAT |
| Lrp2-6S23 | GGGTGGTCGaaTTCTGGTTTTCCACATTTTCTGGTA | TCAGAAGGATCTATGGACCTTCCATaaTCGAAGTCGTAT |
| Lrp2-7S23 | GGGTGGTCGaaGGCTGGCTTTGGCTCTGGAACTATC | GCCCTGAGTTTCATCAGCTCCAGGGaaTCGAAGTCGTAT |
| Lrp2-8S23 | GGGTGGTCGaaGTCTGCTTGGGTTTTCGTTTGAAGA | GCATAGATTGGATTTTCGAAGTTCGaaTCGAAGTCGTAT |
| Lrp2-9S23 | GGGTGGTCGaaTTCCTTTTGCTCAGTGTCCATCTCT | AGATGGAGGGGGAGCCACAGCCACAaaTCGAAGTCGTAT |
| Lrp2-10S23 | GGGTGGTCGaaTTGAAGCTTTAGCAGGAAGAGAAGG | CAGTGTAGCCTGGAGTCGAACTTCTaaTCGAAGTCGTAT |
|  |  |  |
| Oxytocin receptor |  |  |
| Oxtr-1P1S41 | GCTCGACGTaaGCGATGCTCAGGTGCTTCATGAAAA | ACCTGGAACACGGCCACCACCAGGTaaTCCTTTGCAACA |
| Oxtr-2P1S41 | GCTCGACGTaaGACCAGACGACACAGCAGGTCGGGC | GAACATGCCCACCACCTGCAAGTATaaTCCTTTGCAACA |
| Oxtr-3P1S41 | GCTCGACGTaaCGCGCAGCGAGAAAATGTGCACCTG | AGCAATCGAAGACGCCGTCCGCCACaaTCCTTTGCAACA |
| Oxtr-4P1S41 | GCTCGACGTaaCGTGACGTAGGCCTTGGGTCCCCAG | TACAATGTAGACGGCGAGCGTGATCaaTCCTTTGCAACA |
| Oxtr-5P1S41 | GCTCGACGTaaAAGCTGATGAGACCATAGCAGGCGG | TTGAGTCGCAGATTCTGCCAGATCTaaTCCTTTGCAACA |
| Oxtr-6P1S41 | GCTCGACGTaaACACTACTGACCCGTGCCAACGCCG | CGGATTTTGGCCTTGGAGATAAGCTaaTCCTTTGCAACA |
| Oxtr-7P1S41 | GCTCGACGTaaAATGATGAAGGTCATCTTCACTGTG | CGTCCAGCACACGATGAAGGCCAGAaaTCCTTTGCAACA |
| Oxtr-8P1S41 | GCTCGACGTaaATGAAGGCAGAAGCTTCTTTGGGCG | TTGAGGCTGGCCAAGAGCATGGCAAaaTCCTTTGCAACA |
| Oxtr-9P1S41 | GCTCGACGTaaTGCACGAGTTCGTGGAAGAGATGGC | TACCGAGCAGAGCAGCAGAGGAAGCaaTCCTTTGCAACA |
| Oxtr-10P1S41 | GCTCGACGTaaTGCTAATGCTCGTCTCTCCAGGCCG | GGACGAAGGTGGAGGAGTTGCTTTTaaTCCTTTGCAACA |
| Oxtr-11P1S41 | GCTCGACGTaaCTCTGACTCGAGCTGCGACGACTCA | CATGCCGAGGATGGTTGAGAACAGCaaTCCTTTGCAACA |
| Oxtr-12P1S41 | GCTCGACGTaaCAGCCACCAGAGGGGAAGATCGGCG | AATAGGCACCATATACAGCTCCATGaaTCCTTTGCAACA |
| Oxtr-13P1S41 | GCTCGACGTaaACCAGCCCAAGGACAGGAGGGATGC | CCAAGTCAGAATATGACACAGAATCaaTCCTTTGCAACA |
| Oxtr-14P1S41 | GCTCGACGTaaTCTCCCATGGAGACATTTCCCTACC | TCCTCTGATGGCTGAGTGACCCTGTaaTCCTTTGCAACA |
| Oxtr-15P1S41 | GCTCGACGTaaTAGAAATAGGTGGGAGTCCAGGTGA | TGCCCAGGAACACTTGAGTCAGGGTaaTCCTTTGCAACA |
| Oxtr-16P1S41 | GCTCGACGTaaGTGAGGTGCAGGAGAATCTACCCAC | GATAAACAAGGGGTGGAGGGGACAGaaTCCTTTGCAACA |
| Oxtr-17P1S41 | GCTCGACGTaaTGTGCCGTCTTTCACAAGATACCAG | CTCAGGTAACTGGTCACCAATCCTAaaTCCTTTGCAACA |
| Oxtr-18P1S41 | GCTCGACGTaaTCCTTGTCTCATCCCACTGGACTTC | CTCTGCGTCTGCAGCCTCATTTCCCaaTCCTTTGCAACA |
| Oxtr-19P1S41 | GCTCGACGTaaTCAGGTCAGAAGGAAATTAGCACCA | AAGGATGCTAAGATCTGTCCACACAaaTCCTTTGCAACA |
| Oxtr-20P1S41 | GCTCGACGTaaCTCCAAATGCTTTCTGGGATGTCCT | CAGGTGGCTTCACGGCTCAGTTTCCaaTCCTTTGCAACA |
|  |  |  |
| Proenkephalin |  |  |
| Penk-1S23 | GGGTGGTCGaaCTGGAGGTATCCTATCTTCCCACGG | AGTGATGCCTGGGACTATTCTATCTAATCGaaGTCGTAT |
| Penk-2S23 | GGGTGGTCGaaGCCTCCGTACCGTTTCATGAAGCCT | ATATAGCTCGTCCATCTTCTTCATGAATCGaaGTCGTAT |
| Penk-3S23 | GGGTGGTCGaaCATCCTTCTTCATGAAGCCGCCATA | AGTTGGCCAAGGTGTCTCCCTCATCAATCGaaGTCGTAT |
| Penk-4S23 | GGGTGGTCGaaTGTTGGTGCTCTCTTGTTGGTGGCT | CATACCTCTTGCTCATGTCTTCGTCAATCGaaGTCGTAT |
| Penk-5S23 | GGGTGGTCGaaCATTGACAAGGCAGTTGCTCATGGG | CACAAAGCAGCATGTGACAAGAAACAATCGaaGTCGTAT |
| Penk-1A161 | GGTACGCGAaaCTGGAGGTATCCTATCTTCCCACGG | AGTGATGCCTGGGACTATTCTATCTaaAGGTAGGTGTAA |
| Penk-2A161 | GGTACGCGAaaGCCTCCGTACCGTTTCATGAAGCCT | ATATAGCTCGTCCATCTTCTTCATGaaAGGTAGGTGTAA |
| Penk-3A161 | GGTACGCGAaaCATCCTTCTTCATGAAGCCGCCATA | AGTTGGCCAAGGTGTCTCCCTCATCaaAGGTAGGTGTAA |
| Penk-4A161 | GGTACGCGAaaTGTTGGTGCTCTCTTGTTGGTGGCT | CATACCTCTTGCTCATGTCTTCGTCaaAGGTAGGTGTAA |
| Penk-5A161 | GGTACGCGAaaCATTGACAAGGCAGTTGCTCATGGG | CACAAAGCAGCATGTGACAAGAAACaaAGGTAGGTGTAA |
|  |  |  |
| Periostin |  |  |
| Postn-1S72 | CGGTGGAGTaaTCGGTGAAAGTGGTTTGCTGTTTTC | GATGCCAAGCCTAATTGGGCTACCAaaGGCAAGTAGGAT |
| Postn-2S72 | CGGTGGAGTaaTGGCACCATTCCTTCCCTGCTTGCT | GTTGGATGATTTCTCGGAATATGTGaaGGCAAGTAGGAT |
| Postn-3S72 | CGGTGGAGTaaGCCGCAGCTTGTCGTGCAGGGATTT | TGAGGAAGATGCTAAAGCGCTTGTCaaGGCAAGTAGGAT |
| Postn-4S72 | CGGTGGAGTaaTGTTTTGGAGAGCATTTTTATCCCC | CCCCTGGGGTCAGGTGATAAAGAATaaGGCAAGTAGGAT |
| Postn-5S72 | CGGTGGAGTaaTGACTCCGGGTTCGAATCCCTTTCC | TTCCCTGTGTGGTCTTCAGGATATTaaGGCAAGTAGGAT |
| Postn-6S72 | CGGTGGAGTaaAATAGGCTGAAGACTGCCTTGAATG | CGTCATTGCAGGTCCTTCCGTTTTGaaGGCAAGTAGGAT |
| Postn-7S72 | CGGTGGAGTaaCCTGAAGTCGGGATCACCTTCAATT | TGTCACCGTTTCGCCTTCTTTAATCaaGGCAAGTAGGAT |
| Postn-8S72 | CGGTGGAGTaaTCCTGTGGAAATCCTGGTATATTTT | CTGCAAGGTCTCTCCTGTTTCTCCAaaGGCAAGTAGGAT |
| Postn-9S72 | CGGTGGAGTaaTGTGACCTTGGAGACCTCTTTTTGC | GTGACCATCGCCACCTTCAATGAACaaGGCAAGTAGGAT |
| Postn-10S72 | CGGTGGAGTaaCCTGAAGCAGTCTTTTAATCTCCTC | CTGGTATCTTCTTTGCAGGTGTGTCaaGGCAAGTAGGAT |
| Postn-11S72 | CGGTGGAGTaaCTTCTAGGCCCTTGAACCCTTTTGT | CACTGAGAACGGCCTTCTCTTGATCaaGGCAAGTAGGAT |
|  |  |  |
| Uromodulin |  |  |
| Umod-1A161 | GGTACGCGAaaGCATCCAGGTCAAAGGGATCCCCAT | ACCAGGAGGTTACCATCATTACCAGaaAGGTAGGTGTAA |
| Umod-2A161 | GGTACGCGAaaCTTCTGTTGAGTTACTGGCTTCAGC | TGTTGTGGCATTCAGAACACCGTCTaaAGGTAGGTGTAA |
| Umod-3A161 | GGTACGCGAaaGCACGTTGTGACCACACCATCCACC | ATCACCAGTGAAGCCGGTCTGGCAGaaAGGTAGGTGTAA |
| Umod-4A161 | GGTACGCGAaaAGCACACTCATCCATGTCCTCACAC | GTTGGAGCAGTTGTGAGTCCATGGGaaAGGTAGGTGTAA |
| Umod-5A161 | GGTACGCGAaaCCTGACAGGAGCACTTAAACGAGCC | TCAGCTCAGGCGTCAGACGAAAACCaaAGGTAGGTGTAA |
| Umod-6A161 | GGTACGCGAaaCCTGCTCTGAGCACTCATCCACATC | TGGCCAGGGCATGACAGTTACTGAGaaAGGTAGGTGTAA |
| Umod-7A161 | GGTACGCGAaaCGCACAAGTAGTCGCCTTCTGTGTT | CATCCCCTGTAAAGCCCTCGGGACAaaAGGTAGGTGTAA |
| Umod-8A161 | GGTACGCGAaaCTTGACACACCAGCTTTCCATCCGG | TCAGGGTCTCATATGTATTGCAGGGaaAGGTAGGTGTAA |
| Umod-9A161 | GGTACGCGAaaCAGTAAGCCAGATTGCACTCAGGGG | GTCCCCTCCACGGAACTAGGATCGGaaAGGTAGGTGTAA |
| Umod-10A161 | GGTACGCGAaaATGCAATCTTCATCTACCCTGCATT | TGGCAGCGCCATCTGCCGTTATCCGaaAGGTAGGTGTAA |
| Umod-11A161 | GGTACGCGAaaGAGACATCTGTGATGTTGGAGTCCT | CCACACTCCAGCCTGTACTCCAATTaaAGGTAGGTGTAA |
| Umod-12A161 | GGTACGCGAaaCACTTTCTGAGGGACATCTTGATGT | TTCATAAAGCCCAAACTCTGTAGCTaaAGGTAGGTGTAA |
| Umod-13A161 | GGTACGCGAaaGAGCATTGTCTGTCATTCAGGTACA | TCTCGTTCATCACTCTCACTGAAGCaaAGGTAGGTGTAA |
| Umod-14A161 | GGTACGCGAaaCACGGGTCTGATCTATGAAGTTCCC | GTCGTGTTATGGGACCCAAGTTCAGaaAGGTAGGTGTAA |
| Umod-15A161 | GGTACGCGAaaCCTCAAGTTGCTGGAAGCAGCCTTG | AAACAACAGCAGCCAGATGCTCAGGaaAGGTAGGTGTAA |
|  |  |  |
| Vesicular Glutamate Transporter 1 |  |  |
| VG1-1S10 | CGTCGGATGaaGAGCTTGGCGCTCTCCCCGATGGCA | TGTGTTAAACTTCGTAACAGGGTTCaaAGCCCATTAGAT |
| VG1-2S10 | CGTCGGATGaaGGCCAACAAAGCCACACTTCTCCTC | TTTCGTCACTGCCAGCCAGCTGGTCaaAGCCCATTAGAT |
| VG1-3S10 | CGTCGGATGaaCTGGAAACTGCCCCACAGTGGGAGG | AGGCTAGAGGTGTATGGAGTGGAAGaaAGCCCATTAGAT |
| VG1-4S10 | CGTCGGATGaaGGGAGGAGTGGGGTTCCTCGACACT | AGGACTTGCATCTTAAGCCTGAGGCaaAGCCCATTAGAT |
| VG1-5S10 | CGTCGGATGaaAACTGCTAGTGTGCAGCTTCACCCC | CTTTCAGGGGAGTCTGGGTATCCTTaaAGCCCATTAGAT |
| VG1-6S10 | CGTCGGATGaaACAGGCAGAAACAAGCGGAGAACGA | GCCCTCAAAGGGGAGATTTGAGCCCaaAGCCCATTAGAT |
| VG1-7S10 | CGTCGGATGaaAAGAGGTTGAACTGTCCCTCCAAAT | GGTGAAACCTCAAAACCACAAGAGAaaAGCCCATTAGAT |
| VG1-8S10 | CGTCGGATGaaTAGCCTGAGAATCCCTGGGGTCTTG | TAGGGGACCACCTGAATAATCTCGGaaAGCCCATTAGAT |
| VG1-9S10 | CGTCGGATGaaGTGAGAATCAGTCCTGGGAACGGCG | AACCGCCATTTTCCCTCAGAAACGCaaAGCCCATTAGAT |
| VG1-10S10 | CGTCGGATGaaTCCCTGGACAATGATTGTACTAAGC | GGGGTGAGCAGAGCCATTTTGGCCAaaAGCCCATTAGAT |
| VG1-11S10 | CGTCGGATGaaCCACTACTGAGACCTGAAAACTGAG | AGCCGCTGAATTAATAGCTTTGGGCaaAGCCCATTAGAT |
| VG1-12S10 | CGTCGGATGaaAATGGCCACTGAGAAACCGAGGGCG | CATTTAGCCCCTGAGGGACACACAAaaAGCCCATTAGAT |
| VG1-13S10 | CGTCGGATGaaAGGGGAAGCACCCCAGATTTGGAAT | TTCTACGTTGAGAGAGGGGTGTCTAaaAGCCCATTAGAT |
| VG1-14S10 | CGTCGGATGaaCCTGCCCCAGGAACAACCCTCTCCT | TCACAGAGACAGACACCAAGACACGaaAGCCCATTAGAT |
| VG1-15S10 | CGTCGGATGaaTGGGGTGGGGCAGGGCAGGATTTAC | TCCGTACACCAGAGCGTTTATTGGGaaAGCCCATTAGAT |

Table S2. Target mRNA and number of probe pair

| Target mRNA | Number of probe pair |
| --- | --- |
| *Albumin* | 18 |
| *Cytochrome P450 2E1* | 12 |
| *Dopamine receptor D1* | 20 |
| *Dopamine receptor D2* | 21 |
| *Estrogen receptor 1* | 20 |
| *LDL receptor-related protein 2* | 10 |
| *Oxytocin receptor* | 20 |
| *Proenkephalin* | 5 |
| *Periostin* | 11 |
| *Uromodulin* | 15 |
| *Vesicular Glutamate Transporter 1* | 15 |
